# Dietary Vitamin Intake and Cognitive Performance in Adults with Long COVID: The BioICOPER Study

**DOI:** 10.3390/nu18203370

**Published:** 2026-10-14

**Authors:** David Arjol, Silvia Arroyo-Romero, David Rodriguez García, Elena Navarro-Matías, Alicia Navarro-Cáceres, Nuria Suárez-Moreno, Andrea Domínguez-Martín, Manuel A. Gómez-Marcos, Marta Gómez-Sánchez, Leticia Gómez-Sánchez, BioICOPER Investigators Group

**Affiliations:** 1 Unidad de Investigación de Atención Primaria de Salamanca (APISAL), Avd. Portugal, 37005 Salamanca, Spain; silvia_ar@usal.es (S.A.-R.); enavarro@saludcastillayleon.es (E.N.-M.); alicia.nav@usal.es (A.N.-C.); nuria.suarez@usal.es (N.S.-M.); andreadm@usal.es (A.D.-M.); apisal@gmail.com (BioICOPER Investigators Group); 2 Instituto de Investigación Biomédica de Salamanca (IBSAL), Paseo de San Vicente, 37007 Salamanca, Spain; 3 Red de Investigación Sobre Cronicidad, Atención Primaria y Promoción de la Salud (RICAPPS), 37005 Salamanca, Spain; 4 Servicio de Salud de Castilla y León-SACYL, Gestión Regional de Salud, 37005 Salamanca, Spain; 5 Departamento de Medicina, Facultad de Medicina, Universidad de Salamanca, Calle Alfonso X El Sabio s/n, 37007 Salamanca, Spain; 6 Servicio de Hospitalización Domiciliaria, Hospital Universitario Marqués de Valdecilla, 39008 Santander, Spain; martagmzsnchz@gmail.com; 7 Servicio de Urgencias, Hospital Universitario de La Paz, P. de Castellana, 261, 28046 Madrid, Spain; leticiagmzsnchz@gmail.com

**Keywords:** long COVID, post-COVID-19 condition, cognitive performance, brain fog, dietary vitamin intake, vitamin B3, niacin, Montreal Cognitive Assessment

## Abstract

**Background:** Long COVID (LC) is associated with persistent cognitive symptoms, frequently described as brain fog, and with inflammatory, immunometabolic, and energetic mechanisms potentially involved in cognitive functioning. Vitamins participate in biological processes relevant to brain function, but the relationship between habitual dietary vitamin intake and cognitive performance in LC remains poorly studied. **Objective:** To analyze the relationship between dietary vitamin intake and cognitive performance in adults with LC, considering both objective performance and self-reported cognition. **Methods:** A cross-sectional study was conducted in 304 adults with LC. Vitamin intake from foods and beverages was estimated using the EVIDENT seven-day dietary record, previously compared with a validated food-frequency questionnaire. Objective cognitive performance was assessed using normative scores from the Montreal Cognitive Assessment (MoCA) and the Memory Index Score (MIS), as well as MoCA domains and items. Self-reported cognition was assessed using the 30-item Memory Failures of Everyday Questionnaire (MFE-30) and the cognitive subscale of the Modified Fatigue Impact Scale (MFIS). Primary global associations were examined using multiple linear regression with HC3 robust standard errors, whereas bounded MoCA domains were analyzed using proportional-odds ordinal regression and binary item-level outcomes using logistic regression. Benjamini–Hochberg correction was applied within prespecified families of contrasts. **Results:** No associations between vitamin intake and global normative cognitive performance remained significant after false discovery rate (FDR) correction. Median MoCA and MIS scores were 24.00 (IQR, 21.00–26.00) and 11.00 (IQR, 9.00–13.00), respectively. Normative MoCA and MIS scores showed medians of −0.32 (IQR, −0.82 to 0.19) and −0.32 (IQR, −0.76 to 0.14), respectively. Most participants (95.1%) met the dietary reference value for vitamin B3. In secondary ordinal analyses, higher vitamin B3 intake was associated with better MoCA language performance in the fully adjusted model (OR per 1 SD = 2.18, 95% CI 1.45–3.27, *p* < 0.001, *q* = 0.0116). No associations remained significant after FDR correction for everyday cognitive complaints, cognitive fatigue, or MoCA Memory and MoCA Multiple scores. **Conclusions:** In adults with LC, the primary analyses did not identify FDR-significant associations between habitual dietary vitamin intake and global normative cognitive performance. The secondary vitamin B3–language association requires independent confirmation and does not establish a cognitive benefit of higher intake or supplementation. These findings require confirmation in longitudinal studies incorporating nutritional biomarkers, information on supplementation, and broader neuropsychological assessment.

## 1. Introduction

Long COVID (LC) is a complex post-acute condition characterized by the persistence of symptoms following SARS-CoV-2 infection that cannot be explained by an alternative diagnosis [1,2]. Its clinical presentation is heterogeneous and may include fatigue, dyspnea, pain, sleep disturbances, affective symptoms, functional limitations, and persistent cognitive symptoms [3,4]. Within this spectrum, so-called “brain fog” is one of the most disabling manifestations reported by individuals with LC and is commonly described as difficulty concentrating, slowed thinking, memory failures, word-finding difficulties, and reduced efficiency in cognitively demanding activities. These difficulties may interfere with everyday functioning, return to work, social functioning, and quality of life [5,6]. Recent systematic reviews and meta-analyses indicate that these manifestations are frequent, with pooled estimates of approximately 30–32% for brain fog and around 24–28% for persistent memory, concentration, or broader cognitive difficulties, although prevalence varies according to case definition and assessment method [7,8,9,10].

The interpretation of cognitive symptoms in LC remains complex. Available evidence points to several partially overlapping mechanisms, including persistent inflammation, immunometabolic alterations, endothelial or microvascular dysfunction, blood–brain barrier disruption, oxidative stress, and alterations in energy metabolism [11,12]. Recent studies have also shown that cognitive symptoms do not occur in isolation but rather within broader clinical profiles characterized by differing burdens of affective, physical, functional, and quality-of-life impairment [13]. This heterogeneity suggests that cognitive dysfunction in LC may arise from multiple, partially overlapping mechanisms. Because several nutrients participate in mitochondrial energy metabolism, redox regulation, immune signaling, and neuronal function, these pathophysiological alterations provide a rationale for investigating potentially modifiable nutritional factors in LC, without implying an established therapeutic effect of nutritional intervention [14,15].

Among these factors, diet and nutritional status have received increasing attention. Vitamins are involved in processes related to energy metabolism, mitochondrial function, redox regulation, immune responses, neurotransmitter synthesis, and the maintenance of neuronal integrity [16,17]. Within the B-vitamin group, several vitamins contribute to energy production, methylation reactions, and homocysteine metabolism, with potential implications for brain function [16,18,19]. Thiamine and riboflavin act as cofactors in cerebral energy metabolism, whereas vitamins B6, B9, and B12 participate in one-carbon metabolism, methylation, and homocysteine regulation, pathways implicated in cognitive aging [16,19]. Vitamin A, through retinoic-acid signaling, contributes to synaptic plasticity and memory-related processes [20]; vitamin C has antioxidant and neuromodulatory functions [21]; and vitamin D has been linked to neuroimmune and neuroprotective pathways [22]. Vitamin B3, in particular, serves as a precursor of NAD^+^ and NADP^+^, coenzymes involved in mitochondrial processes, redox signaling, and cellular repair [16,23]. However, mechanistic evidence involving NAD^+^ precursors, supplementation, or low nutrient status should not be assumed to imply additional cognitive benefit from higher habitual food-derived niacin-equivalent intake when dietary reference values are already met. Antioxidant vitamins such as vitamin C and fat-soluble vitamins such as vitamins A and D may also participate in immunoinflammatory and oxidative processes [24,25]. Evidence from animal models and from observational and intervention studies in non-COVID populations further supports a possible relationship between vitamin status or intake and cognitive function, although findings differ across vitamins, populations, and study designs and do not demonstrate a uniform cognitive benefit [22,26,27,28].

Clinical evidence regarding micronutrients in general, and vitamins in particular, remains limited and heterogeneous in LC. Available reviews have examined nutritional interventions, vitamin supplementation, multivitamins, nutrient combinations, and dietary patterns, with substantial differences in study design, populations, methods used to assess nutritional exposure, and outcomes evaluated [15,29,30]. Within the BioICOPER cohort, a previous study examined dietary vitamin intake from foods and beverages in relation to central and peripheral arterial stiffness and found no consistent associations between vitamin intake and vascular parameters [31]. Taken together, these findings indicate that research on vitamins in LC remains at an early stage, particularly with regard to cognitive performance.

Outside the context of LC, the literature on vitamins and cognition likewise does not show a consistent pattern. Trials and meta-analyses of B vitamins have shown that reductions in homocysteine are not necessarily accompanied by consistent improvements in global cognition, memory, processing speed, or executive functions, particularly in the absence of clearly established baseline deficiencies [32,33]. Similarly, reviews of vitamin and mineral supplementation in cognitively healthy adults or individuals with mild cognitive impairment have not provided sufficient evidence of sustained cognitive benefit [34,35]. Some observational studies based on dietary intake have reported associations between specific nutrients and performance on multidomain cognitive instruments; however, these findings derive from populations and clinical contexts that differ from LC [36]. For example, higher dietary niacin intake has been associated with lower cognitive decline and better performance on verbal-fluency tasks in older adults, whereas recent trials of NAD^+^ precursors in individuals with cognitive impairment have produced more heterogeneous cognitive results [28,37,38,39].

Cognitive assessment in LC has involved heterogeneous approaches, ranging from comprehensive neuropsychological batteries to brief screening instruments [5,40,41]. Among the latter, the Montreal Cognitive Assessment (MoCA) has been frequently used in post-COVID and LC studies because it provides a multidomain assessment encompassing visuospatial and executive processes, attention, language, memory, and orientation [41,42]. In the Spanish population, available evidence supports its psychometric properties, including internal consistency, criterion validity, and recent evidence regarding its internal structure, and updated normative data are also available [43,44,45].

Despite this growing interest, evidence regarding the relationship between habitual dietary vitamin intake and cognitive performance in adults with LC remains scarce. Moreover, most evidence linking vitamins with cognition derives from older adults, cognitively impaired populations, or neurodegenerative conditions and may not be directly generalizable to LC, given its distinctive combination of immune, vascular, metabolic, autonomic, and symptom-related disturbances [3,4,12,14]. Most of the literature has focused on general symptoms, supplementation, or broader clinical outcomes, without specifically integrating objective cognitive performance and self-reported cognitive complaints. This distinction is relevant because cognitive difficulties in LC may be expressed both through performance on cognitive tests and through the subjective perception of everyday cognitive failures, and these two levels do not always correspond directly.

Accordingly, the present study aimed to analyze the relationship between dietary vitamin intake and cognitive performance in adults with LC. We hypothesized that higher habitual dietary intake of vitamins involved in neurometabolic, antioxidant, and energy-related processes would be associated with better cognitive performance. This relationship was examined at two complementary levels: objective cognitive performance, including global performance, specific domains, and individual items; and self-reported cognition, assessed through everyday cognitive complaints and cognitive fatigue.

## 2. Materials and Methods

### 2.1. Study Design and Participants

The present study is a secondary cross-sectional analysis of the BioICOPER cohort. Participants were assessed at the Primary Care Research Unit of Salamanca (APISAL), Spain, and were recruited between 9 March 2023 and 10 September 2024. The BioICOPER project was registered at ClinicalTrials.gov in April 2023 under the identifier NCT05819840. The study protocol has been published previously [46].

The sample comprised 304 adults diagnosed with Long COVID according to the World Health Organization criteria [1], who were consecutively recruited after providing written informed consent.

Exclusion criteria were terminal illness, inability to attend the primary care center for assessment, a history of cardiovascular disease, or an estimated glomerular filtration rate below 30 mL/min/1.73 m^2^. The participant selection process is shown in Figure 1.

A post hoc minimum detectable-effect sensitivity analysis indicated that, with approximately 80% power and α = 0.05, the study could detect an incremental effect of approximately *f*^2^ = 0.028–0.029, corresponding to a partial *R*^2^ of approximately 0.027–0.028.

The study followed the STROBE recommendations for observational studies [47]. The STROBE checklist is provided as Supplementary Table S1.

### 2.2. Variables and Measurement Instruments

Study assessments were conducted following previously standardized procedures. Before the start of data collection, the healthcare professionals responsible for data collection received specific training to standardize instrument administration, clinical measurements, and data recording.

Information collected during the baseline assessment was entered into an electronic database specifically designed for the project using REDCap (Research Electronic Data Capture), hosted at the Biomedical Research Institute of Salamanca (IBSAL). The platform was used for study data entry, storage, and management. Data quality review and oversight of record consistency were performed by an independent researcher [46].

### 2.3. Dietary Intake Assessment

Dietary intake was recorded using the EVIDENT mobile application [48], which was previously compared with a validated food-frequency questionnaire in Spanish adults, and registered under intellectual property number 00/2014/2207. For seven consecutive days, participants in the BioICOPER study recorded all foods and beverages consumed, including portion sizes and preparation methods. The application classifies foods into predefined groups and allows daily nutrient intake to be estimated using the Spanish food composition tables incorporated into the tool. For micronutrients, the published correlations between EVIDENT and the food-frequency questionnaire during the first week of recording ranged from *r* = 0.092 to *r* = 0.262; vitamin-specific coefficients are provided in Supplementary Table S2.

Based on the seven-day dietary record, daily intakes of vitamin A, B-complex vitamins, vitamin C, and vitamin D were estimated and expressed in the corresponding units for each micronutrient [48]. Vitamin A was expressed as retinol equivalents (RE), whereas folate adequacy was evaluated using dietary folate equivalents (DFE). Vitamin B3 intake was expressed as niacin equivalents (NE); 1 mg NE corresponds to 1 mg of preformed niacin or the niacin equivalent derived from 60 mg of dietary tryptophan. Adequacy of vitamin intake was assessed by comparing the estimated mean daily intake with dietary reference values for adults, using sex-specific cut-off values when applicable [49,50,51]. Participants were considered to meet the corresponding dietary reference value if they consumed at least 750 µg/day of retinol equivalents in men and 650 µg/day in women for vitamin A; 1.2 and 0.9 mg/day for thiamine; 1.6 and 1.3 mg/day for riboflavin; 16 and 14 mg/day for niacin equivalents; 1.7 and 1.6 mg/day for vitamin B6; and 110 and 95 mg/day for vitamin C, respectively. For vitamins with common recommendations for both sexes, the cut-off values were 330 µg/day of dietary folate equivalents for vitamin B9, 4.0 µg/day for vitamin B12, and 15 µg/day for vitamin D.

### 2.4. Cognitive Measures, Fatigue, and Affective Symptoms

To capture complementary dimensions of cognitive functioning, the assessment included measures of global objective performance, episodic memory, everyday cognitive complaints, and perceived cognitive fatigue. Cognitive performance was assessed using the official Spanish version 8.1 of the Montreal Cognitive Assessment (MoCA) [52]. The MoCA is a brief cognitive assessment instrument comprising tasks that assess visuospatial/executive functioning, naming, attention, language, abstraction, memory, and orientation, with a total score ranging from 0 to 30 points. The Memory Index Score (MIS) is a specific index of episodic memory derived from the MoCA memory domain, with a score ranging from 0 to 15 points, and can be used as an independently scored subtest of the instrument. In the Spanish population, available evidence has shown adequate psychometric properties of the MoCA [43]. In a Spanish community sample, a 12-item internal structure organized into two correlated factors, MoCA Memory and MoCA Multiple, has also been described [44]. Updated normative data for both the MoCA and MIS are also available for Spanish adults [45].

Everyday cognitive complaints were assessed using the 30-item Memory Failures of Everyday Questionnaire (MFE-30) [53]. Cognitive fatigue was assessed using the cognitive subscale of the Modified Fatigue Impact Scale (MFIS) [54]. Anxiety and depressive symptoms were assessed using the Spanish version of the Goldberg Anxiety and Depression Scale (GADS) [55,56]. Higher scores indicate greater impairment on the MFE-30, MFIS, and GADS. Cognitive assessments were completed before nutrient intakes were derived from the dietary records; therefore, the assessors administering the cognitive instruments were unaware of participants’ subsequently calculated vitamin intake.

### 2.5. Sociodemographic, Clinical, and Lifestyle Variables

Sociodemographic, clinical, and lifestyle variables were collected using standardized questionnaires, study-specific forms, and review of medical records. Age, sex, educational level, and duration of LC were recorded. Duration of LC was calculated as the interval, in months, between the onset of persistent symptoms and the baseline assessment. Hospitalization during the acute phase of COVID-19 was recorded as an indicator of acute disease severity. Long COVID symptom burden and persistent taste or olfactory dysfunction were also recorded.

Body weight was measured using an InBody 230 body composition analyzer (InBody Co., Ltd., Seoul, Republic of Korea), and height was measured using a Seca 222 stadiometer (seca GmbH & Co. KG, Hamburg, Germany), with participants barefoot. Body mass index (BMI) was calculated as weight (kg)/height (m)^2^, and obesity was defined as BMI ≥ 30 kg/m^2^. Systolic and diastolic blood pressure were measured in accordance with the recommendations of the European Society of Hypertension [57], and mean arterial pressure was calculated from these values. Hypertension was defined as blood pressure ≥ 140/90 mmHg, a previous diagnosis, or current antihypertensive treatment.

Fasting blood samples were obtained to determine fasting glucose, total cholesterol, HDL cholesterol, LDL cholesterol, and triglycerides. Diabetes mellitus was defined as fasting glucose ≥ 126 mg/dL, a previous diagnosis, or current antidiabetic treatment. Dyslipidemia was defined as a previous diagnosis, lipid-lowering treatment, or an abnormal lipid profile at assessment.

Current pharmacological treatment was obtained using a standardized data collection form and review of medical records. The use of psychotropic medications, including antidepressants, benzodiazepines, and hypnotics, was recorded in accordance with the previously published BioICOPER protocol [46]. The number of SARS-CoV-2 vaccine doses received before the baseline assessment was also recorded. Dietary supplement use was also recorded.

Alcohol consumption was recorded in grams per week based on self-reported intake during the previous seven days. Tobacco use was assessed using a standardized questionnaire adapted from the WHO MONICA project [58]. Adherence to the Mediterranean diet was assessed using the 14-item Mediterranean Diet Adherence Screener (MEDAS), with higher scores indicating greater adherence to the Mediterranean dietary pattern [59]. Physical activity was assessed using the Global Physical Activity Questionnaire (GPAQ) and expressed as metabolic equivalent minutes per week [60]. In addition, physical activity was objectively recorded over seven days using an Omron HJ-321-E digital pedometer (Omron Healthcare Co., Ltd., Kyoto, Japan), a model previously evaluated for step-count validity [61], which provided total steps, aerobic steps, distance traveled, and estimated energy expenditure.

### 2.6. Statistical Analysis

The distribution of continuous variables was assessed using the Shapiro–Wilk test, skewness and kurtosis coefficients, and graphical inspection of histograms, box plots, and quantile–quantile plots. Continuous variables were described as mean ± standard deviation or median and interquartile range, according to their distribution, and categorical variables as absolute frequencies and percentages. Descriptive comparisons by sex were performed using Student’s *t*-test, with Welch’s correction when appropriate, or the Mann–Whitney *U* test for continuous variables, and the χ^2^ test or Fisher’s exact test for categorical variables.

Missing data were examined by variable and by model. Multivariable models were estimated using complete cases. Participants included in the primary fully adjusted normative cognitive models were compared with those excluded because of incomplete data. Multiple imputation was additionally performed as a sensitivity analysis.

Nutritional adequacy was described for descriptive purposes only as the proportion of participants meeting the dietary recommendations for each vitamin. Associations between vitamin intake and cognitive indicators were estimated using separate models for each micronutrient, with each vitamin entered as a continuous exposure. Vitamins were not entered simultaneously in the same model because the study aimed to estimate vitamin-specific associations separately and because mutual adjustment for correlated nutrient intakes may yield unstable or difficult-to-interpret coefficients.

MoCA and MIS scores were transformed into normative z scores according to the reference data available for Spanish adults [45]. Associations with continuous scores were estimated using multivariable linear regression with HC3 robust standard errors. This approach was applied to normative MoCA and MIS scores, the MFE-30, and the cognitive subscale of the MFIS. MoCA domain scores were analyzed primarily using proportional-odds ordinal regression because of their discrete and bounded distributions. Vitamin intakes were standardized, and estimates from ordinal models are reported as odds ratios per 1 SD higher intake. The proportional-odds assumption was assessed using the Brant test. Quasibinomial and linear HC3 models were examined as sensitivity analyses. When item-level outcomes were binary, associations were estimated using logistic regression and reported as odds ratios with 95% confidence intervals. Where applicable, adjusted probabilities were estimated at the 25th and 75th percentiles of the exposure by averaging predictions from the fully adjusted logistic model over the observed covariate distribution of the analytical sample. Absolute probability differences and their 95% confidence intervals were calculated using the delta method, treating the selected exposure levels and observed covariate distribution as fixed.

Two prespecified adjustment models were estimated. For normative MoCA and MIS scores, Model 1 included total energy intake, and Model 2 additionally included BMI and Mediterranean diet adherence. For MoCA domains and items, the MFE-30, and the cognitive subscale of the MFIS, Model 1 included age, sex, educational level, and total energy intake, and Model 2 additionally included BMI and Mediterranean diet adherence. Covariate selection was informed by a directed acyclic graph (Supplementary Figure S1) and prior evidence on demographic, nutritional, metabolic, and Long COVID-related determinants of dietary intake and cognition. Because normative MoCA and MIS scores already incorporate age, sex, and education within the published β-GAM normative procedure [45], these variables were not additionally included in these models; their omission was intended to avoid duplicate demographic adjustment and should not be interpreted as evidence that all possible demographic effects were completely residualized. These variables were included directly in models of non-normative cognitive outcomes.

Sensitivity analyses evaluated the stability of the observed associations through additional adjustment for alcohol and tobacco consumption, anxiety and depressive symptoms, psychotropic medication use, and physical activity. Additional models considered acute-phase hospitalization, Long COVID duration, protein intake, and dietary supplement use, and analyses excluding supplement users were also performed. Alternative nutritional specifications were also evaluated, including winsorized exposure, energy-residualized exposure, and exposure expressed as nutrient density per 1000 kcal. Energy-reporting plausibility was evaluated using the Goldberg approach [62]. In addition, models excluding potentially influential observations according to Cook’s distance, leverage, and studentized residuals were estimated. As an additional sensitivity analysis, models were repeated using the MoCA Memory and MoCA Multiple scores derived from the Spanish two-factor MoCA solution [44].

Model assumptions were evaluated by inspection of residuals, fitted values, and quantile–quantile plots. Multicollinearity was examined using correlation matrices and the variance inflation factor. Influential observations were evaluated using leverage, Cook’s distance, and studentized residuals.

Correction for multiple comparisons was performed using the Benjamini–Hochberg procedure to control the false discovery rate within prespecified families of contrasts: normative MoCA/MIS scores, MoCA domains, and self-reported cognition (MFE-30 and the MFIS cognitive subscale). These families comprised 36, 126, and 36 contrasts, respectively. Associations with *q* values < 0.05 were considered statistically significant after correction for multiplicity. Item-level analyses were conducted, when appropriate, as secondary follow-up analyses of associations observed at the domain level.

All analyses were performed using R version 4.5.0 [63], with a two-sided significance level of α = 0.05. Multiple imputation was implemented using the mice package [64], with estimates pooled according to Rubin’s rules. Model diagnostics were conducted using the car package [65], and robust standard errors were calculated using the lmtest [66] and sandwich [67] packages.

### 2.7. Ethical Considerations

This study was approved on 27 June 2022, by the Ethics Committee for Research with Medicines of the Salamanca Health Area (CEIm; Ref. PI 2022 06 1048). The study was conducted in accordance with the principles of the Declaration of Helsinki and applicable WHO ethical guidance [68,69]. Participant confidentiality was maintained throughout the study in accordance with Organic Law 3/2018 and Regulation (EU) 2016/679 on the protection of personal data. All participants provided written informed consent before inclusion in the study, after receiving detailed information about the study procedures.

### 2.8. Use of Artificial Intelligence Tools

During the preparation of this manuscript, ChatGPT (GPT-5.5; OpenAI) was used to assist with English-language editing. Generative AI was not used to generate data, perform statistical analyses, interpret the results, create scientific images, or alter the study findings. All AI-assisted outputs were checked against the original manuscript and study results by the authors. The authors reviewed and approved all AI-assisted edits and take full responsibility for the final content of the manuscript.

## 3. Results

### 3.1. Participant Characteristics at Study Inclusion

The sample comprised 304 participants, of whom 207 were women (68.1%). Compared with women, men were older (*p* = 0.004) and had a less favorable cardiometabolic profile, with higher blood pressure, fasting glucose, triglyceride, and BMI values, lower HDL-c concentrations, and a higher prevalence of hypertension, diabetes mellitus, and obesity (all *p* ≤ 0.001). Men also reported higher alcohol consumption and a lower frequency of alcohol abstinence (both *p* < 0.001), as well as a greater number of cigarettes smoked per day among participants with available smoking data (*p* = 0.001). They also showed more frequent acute-phase hospitalization (*p* < 0.001), lower anxiety and depressive symptom scores (both *p* < 0.001), and less frequent use of psychotropic medication (*p* = 0.008). Sociodemographic, clinical, and lifestyle characteristics are presented in Table 1.

Median MoCA, MIS, MFE-30, and MFIS cognitive subscale scores were 24.00 (IQR, 21.00–26.00), 11.00 (IQR, 9.00–13.00), 31.50 (IQR, 16.00–53.00), and 19.50 (IQR, 7.00–29.00), respectively. No statistically significant sex differences were observed in MoCA scores (*p* = 0.059), whereas MIS scores and the MoCA delayed recall domain were lower in men (both *p* < 0.001), and MFE-30 and MFIS cognitive subscale scores were higher in women (*p* = 0.002 and *p* = 0.001, respectively). Normative MoCA and MIS scores showed medians of −0.32 (IQR, −0.82 to 0.19) and −0.32 (IQR, −0.76 to 0.14), respectively, with no differences by sex (*p* = 0.475 and *p* = 0.715). Table 2 presents MoCA and MIS scores, their normative scores, MoCA domains, everyday cognitive complaints, and cognitive fatigue. MoCA items and MoCA Memory and MoCA Multiple scores are described in Supplementary Table S3.

### 3.2. Vitamin Intake and Dietary Adequacy

No statistically significant sex differences were observed in the distribution of any of the vitamins analyzed (all *p* > 0.05). However, the proportion of participants meeting dietary reference values was higher among women for vitamin A (*p* = 0.014), vitamin B1 (*p* < 0.001), vitamin B2 (*p* = 0.001), and vitamin B6 (*p* = 0.011). Vitamin B3 intake had a median of 33.16 mg NE/day (IQR, 27.46–38.35), and 95.1% of participants met the corresponding dietary reference value. In contrast, vitamin D had the lowest proportion meeting the dietary reference value (1.3%). Vitamin intake values are presented in Table 3, and the proportion of participants meeting dietary reference values is shown in Figure 2.

### 3.3. Primary Associations Between Vitamin Intake and Normative Cognitive Performance

The comparison between participants included in and excluded from the primary fully adjusted normative cognitive models is presented in Supplementary Table S4. Within the primary family of 36 contrasts, continuous vitamin intake showed no associations with global normative cognitive performance that remained statistically significant after Benjamini–Hochberg correction. Across the primary models, the maximum R^2^ values were 0.039 for the MoCA normative z score and 0.053 for the MIS normative z score (Supplementary Table S6).

Before correction for multiple comparisons, inverse associations were observed between normative MIS scores and vitamin B12 in Model 1, β = −0.011, 95% CI −0.020 to −0.002, *p* = 0.014, and in Model 2, β = −0.011, 95% CI −0.020 to −0.002, *p* = 0.015. Inverse associations were also observed between normative MIS scores and vitamin C in Model 1, β = −0.002, 95% CI −0.004 to −0.001, *p* = 0.008, and in Model 2, β = −0.002, 95% CI −0.004 to −0.001, *p* = 0.010. After FDR correction, none of these associations remained statistically significant. Complete results of the primary models are presented in Table 4, FDR-adjusted q values in Supplementary Table S5, and model diagnostics in Supplementary Table S6.

### 3.4. Secondary Analyses of MoCA Domains and Items

In proportional-odds ordinal models, higher vitamin B3 intake was associated with better performance in the MoCA language domain in both adjustment models. Across the prespecified family of 126 domain-level contrasts, the vitamin B3–language association was the only association that remained statistically significant after FDR correction in both models. Several MoCA domains and items showed substantial ceiling effects, as detailed in Supplementary Table S3.

For each 1 SD increase in vitamin B3 intake, the odds of a higher language-domain score were 2.19 (95% CI 1.46–3.28; *p* < 0.001; q = 0.0116) in Model 1 and 2.18 (95% CI 1.45–3.27; *p* < 0.001; *q* = 0.0116) in Model 2. The Brant tests did not detect evidence against the proportional-odds assumption for the vitamin B3–language models. Quasibinomial and HC3 linear sensitivity estimates, together with additional sensitivity analyses, are reported in Supplementary Tables S7 and S8.

To further characterize the vitamin B3–language association, verbal fluency was examined post hoc using logistic regression. In the fully adjusted model, each 1 SD increase in vitamin B3 intake (approximately 9.14 mg/day) was associated with higher odds of meeting the MoCA verbal-fluency criterion (OR = 2.59, 95% CI 1.61–4.17; *p* < 0.001); the corresponding OR per 5 mg/day increase was 1.69 (95% CI 1.30–2.19). At vitamin B3 intakes of 27.46 and 38.61 mg/day, corresponding to the 25th and 75th percentiles in the analytical sample, the adjusted probabilities of meeting the verbal-fluency criterion were 40.0% and 65.3%, respectively. The absolute difference was 25.2 percentage points (95% CI 14.7–35.8). The item-level analysis was exploratory (Supplementary Table S7 and Figure S2).

### 3.5. Self-Reported Cognition and Sensitivity Analyses

Vitamin intake was not associated with MFE-30 scores or the MFIS cognitive subscale (Supplementary Table S9). Models using MoCA Memory and MoCA Multiple scores showed no associations with vitamin intake after correction for multiplicity (Supplementary Table S10).

In sensitivity analyses of the vitamin B3–language association, the OR per 1 SD was 2.24 (95% CI 1.53–3.28; *p* < 0.001) after additional adjustment for acute-phase hospitalization, Long COVID duration, and GADS anxiety and depression scores, and 2.31 (95% CI 1.56–3.41; *p* < 0.001) after further adjustment for symptom burden and persistent taste/olfactory dysfunction. Additional models adjusting for psychotropic medication use yielded similar estimates.

Vitamin B3 intake was correlated with total energy intake (Spearman ρ = 0.797) and protein intake (ρ = 0.354). Among participants with the data required for Goldberg classification, 38.5% were classified as under-reporters, 60.8% as plausible energy reporters, and 0.7% as over-reporters. Additional adjustment for protein intake, restriction to plausible energy reporters according to the Goldberg approach, adjustment for dietary supplement use, exclusion of supplement users, and multiple-imputation analyses were also performed. Results of these sensitivity analyses are reported in Supplementary Table S8.

## 4. Discussion

The present study examined the relationship between dietary vitamin intake and cognitive performance in adults with LC. Overall, the results do not support a relationship between vitamin intake and global cognitive performance. In secondary ordinal analyses, vitamin B3 intake was associated with better performance in the MoCA language domain after FDR correction in both adjustment models. The post hoc verbal-fluency analysis showed an association in the same direction but remains exploratory and hypothesis-generating. No associations were observed with everyday cognitive complaints, cognitive fatigue, or MoCA Memory and MoCA Multiple scores.

Evidence on micronutrients and cognition in LC remains limited and heterogeneous [29,30,31], and studies in other clinical and community populations have likewise not shown a uniform cognitive benefit. Trials and meta-analyses of B vitamins indicate that homocysteine reduction does not consistently translate into improvement in global cognition or specific domains [32,33], while evidence for vitamins C and D is also heterogeneous [21,22,34,35]. Interpretation must also consider the exposure range in the present sample: 95.1% of participants met the dietary reference value for vitamin B3, whereas only 1.3% met the dietary vitamin D reference value from recorded foods and beverages. The latter is descriptive and cannot be interpreted as biological vitamin D deficiency because supplement exposure was incompletely captured and sun exposure was not assessed.

The observed association between vitamin B3 and the MoCA language domain is compatible with several biologically plausible pathways. Vitamin B3 acts as a precursor of NAD^+^ and NADP^+^, coenzymes involved in energy metabolism, mitochondrial function, redox balance, and cellular repair [16,23]. However, this biological rationale should be interpreted in the context of the exposure observed in the present study. The median vitamin B3 intake was 33.16 mg/day (IQR, 27.46–38.35), and 95.1% of participants met the dietary reference value; therefore, the analyses primarily reflect variation within a range of dietary intake that generally met the reference value rather than a comparison between deficient and sufficient states. Accordingly, evidence derived from NAD-precursor supplementation or low nutritional status is not directly equivalent to the habitual dietary exposure assessed here. Previous observational studies provide more directly comparable evidence: higher dietary niacin intake has been associated with slower cognitive decline and lower Alzheimer disease risk [28], better cognitive performance including the Animal Fluency Test [37], and, more recently, with cognitive performance in NHANES analyses identifying niacin as a prominent B-vitamin contributor [70].

Interpretation of this association nevertheless requires consideration of protein and tryptophan. Vitamin B3 intake was moderately correlated with protein intake in the present sample, and niacin-equivalent intake incorporates the metabolic contribution of tryptophan [71]. Evidence relating dietary protein and specific amino acids to cognition remains heterogeneous: a meta-analysis found no overall association between protein intake and global cognition in older adults [72], while the literature on tryptophan and cognition remains limited and context dependent [71]. The persistence of the B3 estimate after additional adjustment for protein reduces, but does not eliminate, the possibility that correlated dietary exposures contributed to the observed association.

These pathways may be relevant in LC, in which mechanisms related to mitochondrial dysfunction, immunometabolic alterations, and persistent fatigue have been proposed [14,15]. Interest in the NAD pathway in LC has also increased following studies of NAD precursors; in a recent randomized trial, nicotinamide riboside increased NAD^+^ levels but did not produce differences from placebo in the primary cognitive outcomes [73]. Similarly, randomized studies of nicotinamide or nicotinamide riboside in mild cognitive impairment, subjective cognitive decline, and early Alzheimer disease have altered NAD-related measures or biomarkers without demonstrating consistent benefits on conventional cognitive outcomes [38,39,74]. These intervention studies provide mechanistic context but are not directly comparable with habitual dietary niacin-equivalent intake in a population in which most participants met the dietary reference value. This evidence provides a biological context for interpreting the B3–language association but does not allow the observed finding to be attributed to a specific metabolic mechanism or justify supplementation recommendations.

The association with the verbal fluency task is of neuropsychological interest because such tasks occupy an intermediate position between language and executive functioning. Producing words under a time constraint requires lexical access, rapid retrieval, maintenance of task rules, inhibition of irrelevant responses, strategic switching, and monitoring of repetitions [75,76,77,78]. Interpretation of the domain-specific finding should also consider the restricted distributions of several MoCA components. Marked ceiling effects were observed for some items and domains, including naming and clock-contour performance, which reduced the available variability for detecting associations in those outcomes. The language domain therefore provided comparatively greater usable variability, although this does not establish that the B3 association is specific to language. However, because verbal fluency was examined post hoc using a single MoCA item, this finding should be considered exploratory rather than independent confirmation of the domain-level association.

No evidence of association was observed between vitamin intake and MFE-30 scores or the MFIS cognitive subscale. This distinction is relevant in LC, where subjective cognitive complaints may overlap with fatigue, sleep disturbances, affective symptoms, and functional burden and do not always correspond directly to performance on brief cognitive tests [5,79]. In addition, the MFE-30 assesses memory failures in everyday situations, whereas the MFIS captures the perceived impact of fatigue, including its cognitive dimension; therefore, the two instruments assess dimensions that are related to, but not equivalent to, objective cognitive performance [53,54]. The B3 association was also not reproduced for MoCA Memory or MoCA Multiple scores [44], and the inverse nominal associations of vitamins B12 and C with normative MIS scores did not survive FDR correction and should not be interpreted as evidence of detrimental effects. Given their counterintuitive direction and the cross-sectional design, these nominal associations may reflect chance, residual dietary confounding, or reverse causation.

From a clinical perspective, the predominantly null findings do not support supplementation specifically to improve cognition in LC. Because the B3 association occurred largely within an intake range meeting the dietary reference value, it should not be interpreted as evidence that increasing niacin-equivalent intake above recommended levels would improve cognitive performance. The secondary B3–language association and post hoc verbal-fluency finding require independent confirmation**.**

### Strengths and Limitations

Several limitations directly affect interpretation of the findings. The cross-sectional design precludes causal inference and does not exclude reverse causation. Dietary intake was self-reported, and differential recording error is possible if cognitive symptoms, fatigue, or other LC manifestations affected completion of the seven-day record. Although EVIDENT provides detailed dietary information, its validation was against another self-report instrument and vitamin-specific correlations were modest [48]. Dietary records did not quantify vitamin exposure from supplements; supplement-use information was unavailable for 107 participants, and preformed niacin could not be separated from niacin equivalents. Dietary intake also does not necessarily reflect biological vitamin status because no nutritional biomarkers or NAD-related metabolites were available. For vitamin D specifically, sun exposure was not assessed, further limiting interpretation of the low proportion of participants meeting the dietary reference value from foods and beverages alone.

Some LC-specific characteristics were incompletely captured at the individual level, including recruitment pathway, and residual confounding related to disease characteristics may therefore remain despite sensitivity adjustment for acute hospitalization, LC duration, anxiety and depressive symptoms, symptom burden, persistent taste/olfactory dysfunction, and psychotropic medication use. Psychotropic medication itself may contribute residual confounding. Seasonality of dietary intake was not modeled, and residual confounding from unmeasured dietary, socioeconomic, metabolic, or clinical factors cannot be excluded. Complete-case comparisons also showed that included participants were older than those excluded, although most cognitive and dietary variables were broadly similar. In addition, individual-level data were unavailable for persons who declined participation, precluding formal comparison with enrolled participants, and the exclusion of individuals with established cardiovascular disease or severe renal impairment may limit generalizability to the broader Long COVID population. Finally, MoCA is a screening instrument [52]; its bounded domain scores and single-item verbal-fluency measure cannot substitute for comprehensive neuropsychological assessment. Marked ceiling effects in several MoCA domains and items may also have reduced between-participant variability and the ability to detect domain-specific associations.

Strengths include the seven-day dietary record, normative MoCA and MIS scoring, complementary objective and self-reported cognitive outcomes, prespecified multiplicity correction, outcome-appropriate ordinal modeling of bounded MoCA domains, model diagnostics, and extensive sensitivity analyses addressing protein intake, energy-reporting plausibility, LC-related covariates, psychotropic medication, supplement use, and missing data.

## 5. Conclusions

In this cross-sectional study of adults with LC, habitual dietary vitamin intake from foods and beverages showed no consistent relationship with global cognitive performance. A secondary association between higher vitamin B3 intake and better MoCA language performance remained statistically significant after FDR correction in both ordinal adjustment models. The post hoc verbal-fluency finding should be considered exploratory and hypothesis-generating. These findings did not extend to global normative cognitive scores, everyday cognitive complaints, cognitive fatigue, or MoCA Memory and MoCA Multiple scores. Overall, the data do not support a general relationship between vitamin intake and cognitive performance in LC. Because most participants met the dietary reference value for vitamin B3, the secondary association was observed predominantly within a dietary-intake range in which most participants met the reference value and should not be interpreted as evidence that intake above recommended levels or supplementation would improve cognition. The secondary vitamin B3 finding requires independent confirmation in longitudinal studies incorporating nutritional biomarkers and comprehensive neuropsychological assessment.

## Figures and Tables

**Figure 1:**
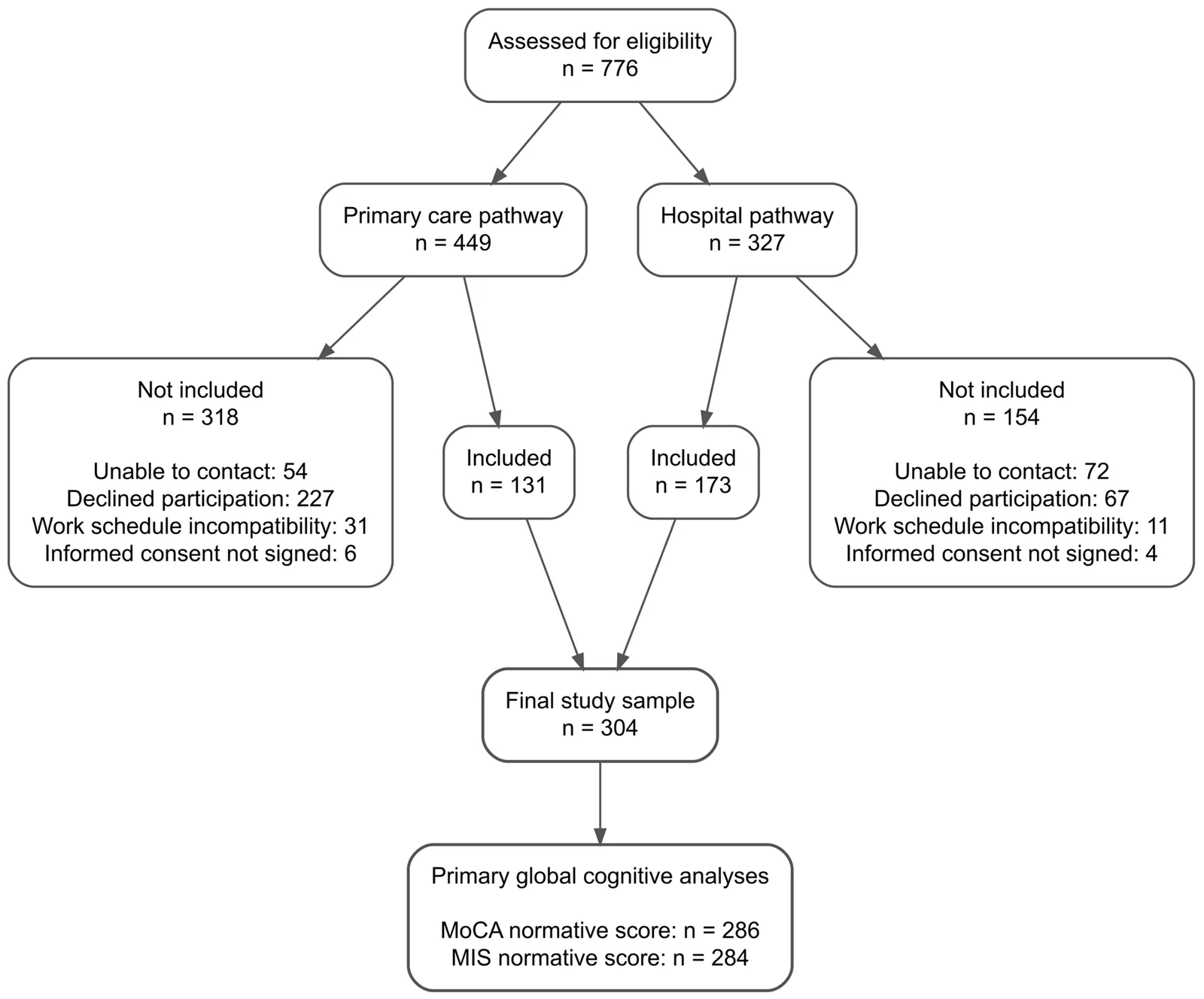
Participant selection process for the BioICOPER study. The flow diagram summarizes the number of participants assessed through each recruitment pathway, the reasons for non-inclusion, the final study sample, and the outcome-specific analytical sample sizes for the primary global cognitive analyses. The analytical sample sizes were *n* = 286 for the MoCA normative score and *n* = 284 for the MIS normative score in both Model 1 and Model 2.

**Figure 2:**
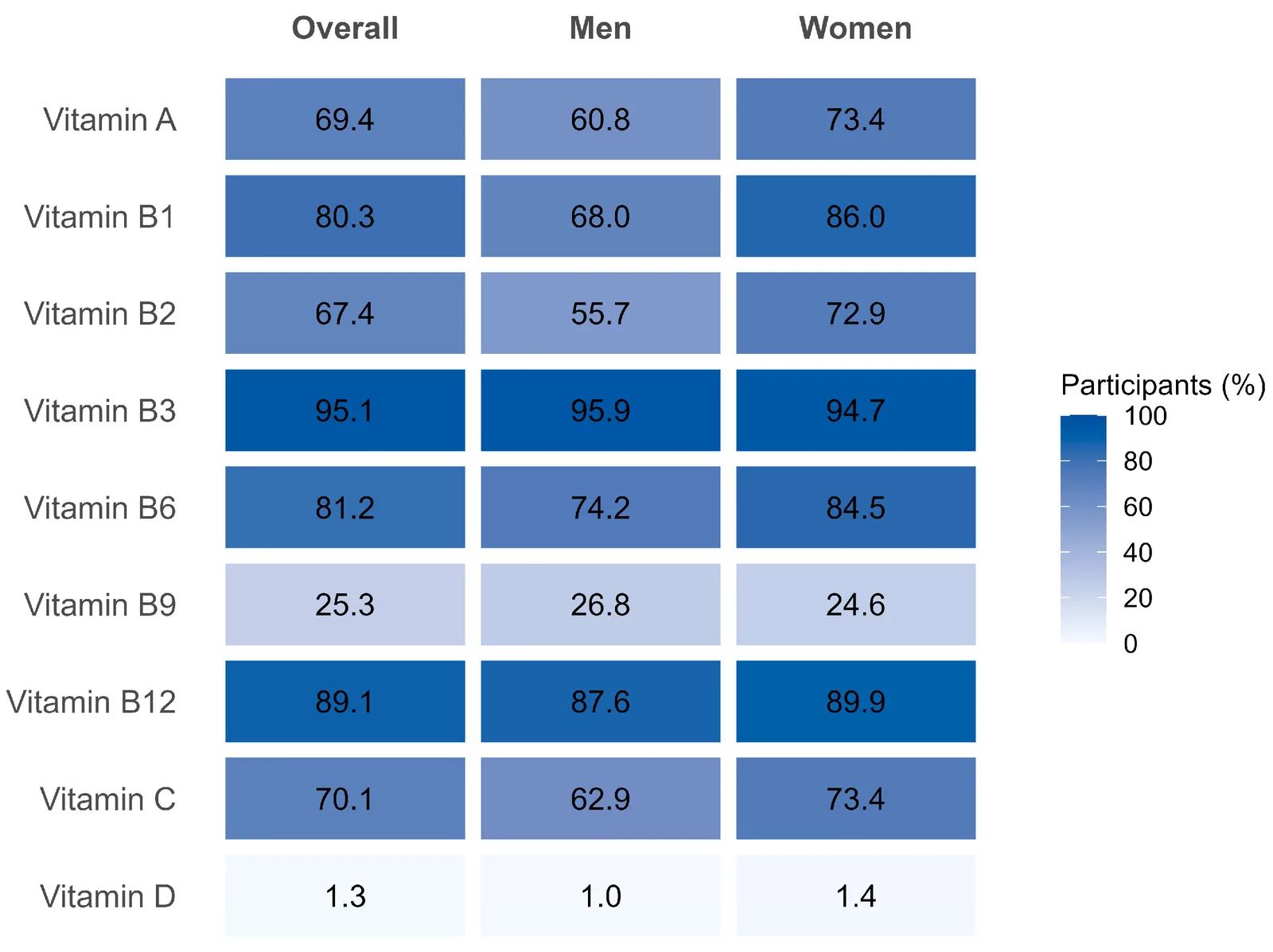
Proportion of participants meeting dietary reference values for each vitamin, overall and by sex. Values inside cells indicate the percentage of participants meeting the corresponding dietary reference value for each vitamin. Percentages represent dietary intake from recorded foods and beverages only; vitamin intake from dietary supplements was not included in these estimates. Adequacy variables were used for descriptive purposes only.

**Table 1:** Sociodemographic, clinical, and lifestyle characteristics of the study population overall and by sex.

Variable	Reference Value or Clinical Cut-Off	Overall (*n* = 304)	Men (*n* = 97)	Women (*n* = 207)	*p*-Value
Age, years	—	52.78 ± 11.91	55.70 ± 12.28	51.41 ± 11.51	0.004
Long COVID duration, months	—	40.23 (34.85–46.17)	40.50 (33.20–46.57)	40.13 (35.63–45.10)	0.991
Educational level: primary, *n* (%)	—	33 (10.9)	14 (14.4)	19 (9.2)	0.295
Educational level: secondary, *n* (%)	—	154 (50.7)	50 (51.5)	104 (50.2)	
Educational level: university, *n* (%)	—	117 (38.5)	33 (34.0)	84 (40.6)	
Alcohol, g/week	—	0.00 (0.00–30.00)	25.00 (0.00–95.00)	0.00 (0.00–20.00)	<0.001
No alcohol consumption, *n* (%)	—	163 (53.6)	36 (37.1)	127 (61.4)	<0.001
MD score	Higher score = greater adherence	8.00 (7.00–9.00)	8.00 (6.00–9.00)	8.00 (7.00–9.00)	0.424
MD adherence, *n* (%)	MEDAS ≥ 9	123 (40.5)	38 (39.2)	85 (41.1)	0.755
Total energy intake, kcal/day	—	1730.12 (1392.05–2036.14)	1781.77 (1373.90–2048.79)	1693.08 (1399.87–2018.63)	0.760
MET-min/week	—	3360 (1680–7320)	3480 (1800–6480)	2880 (1560–8160)	0.246
Number of steps per day	—	6869 (4753–9251)	6041 (4756–8639)	7344 (4753–9503)	0.161
Years of smoking	—	22.00 (14.00–31.00)	27.50 (16.00–34.00)	19.00 (14.00–29.00)	0.074
Current smoker, *n* (%)	Current smoking	16 (5.3)	8 (8.2)	8 (3.9)	0.111
No. of cigarettes/day	—	14.50 (8.00–20.00)	20.00 (10.00–25.00)	10.00 (8.00–20.00)	0.001
Acute-phase hospitalization, *n* (%)	Hospitalization during acute COVID-19	84 (28.2)	42 (44.2)	42 (20.7)	<0.001
GADS anxiety score	Higher score = greater symptoms	4.00 (1.00–7.00)	1.00 (0.00–6.00)	5.00 (1.00–7.00)	<0.001
GADS depression score	Higher score = greater symptoms	4.00 (1.00–6.00)	2.00 (0.00–5.00)	4.00 (2.00–6.00)	<0.001
Any psychotropic medication, *n* (%)	Current use	106 (34.9)	23 (23.7)	83 (40.1)	0.008
Antidepressant use, *n* (%)	Current use	76 (25.0)	14 (14.4)	62 (30.0)	0.006
Benzodiazepine use, *n* (%)	Current use	56 (18.4)	10 (10.3)	46 (22.2)	0.019
Anxiolytic use, *n* (%)	Current use	16 (5.3)	5 (5.2)	11 (5.3)	1.000
Supplement use, *n* (%)	Current use	11 (5.6)	2 (3.5)	9 (6.4)	0.516
SBP, mmHg	<140 mmHg	118.00 (108.00–131.25)	128.50 (119.50–139.00)	112.50 (104.00–126.50)	<0.001
DBP, mmHg	<90 mmHg	76.88 ± 11.12	82.34 ± 11.04	74.33 ± 10.22	<0.001
MAP, mmHg	—	91.25 ± 12.43	98.04 ± 11.33	88.06 ± 11.64	<0.001
Hypertension, n (%)	≥140/90 mmHg, diagnosis, or treatment	109 (35.9)	52 (53.6)	57 (27.5)	<0.001
Total cholesterol, mg/dL	<200 mg/dL	186 (163–207)	182 (159–200)	188 (166–211)	0.073
LDL-c, mg/dL	<100 mg/dL	111 (92–132)	114 (91–131)	110 (93–132)	0.724
HDL-c, mg/dL	≥40 men; ≥50 women	56 (46–66)	46 (40–56)	61 (51–68)	<0.001
Triglycerides, mg/dL	<150 mg/dL	87 (67–121)	104 (82–141)	82 (64–108)	<0.001
Dyslipidemia, *n* (%)	Diagnosis, treatment, or abnormal lipid profile	201 (66.1)	71 (73.2)	130 (62.8)	0.074
FPG, mg/dL	<100 mg/dL	84 (79–91)	88 (83–100)	82 (78–88)	<0.001
Diabetes mellitus, *n* (%)	FPG ≥ 126 mg/dL, diagnosis, or treatment	37 (12.2)	22 (22.7)	15 (7.2)	<0.001
Weight, kg	—	74.65 (61.35–87.55)	87.30 (78.00–94.90)	65.60 (58.60–79.30)	<0.001
Height, cm	—	163.75 (159.00–170.00)	172.50 (167.50–177.00)	161.00 (157.00–165.00)	<0.001
BMI, kg/m^2^	<30 kg/m^2^	27.60 (23.60–31.20)	29.40 (26.00–32.50)	26.40 (22.60–30.60)	<0.001
Obesity, n (%)	BMI ≥ 30 kg/m^2^	99 (32.6)	44 (45.4)	55 (26.6)	0.001
Number of vaccine doses		2.93 ± 1.17	3.11 ± 1.24	2.85 ± 1.13	0.072

Values are presented as mean ± standard deviation or median (interquartile range) for continuous variables and as *n* (%) for categorical variables. Reference values or clinical cut-off points are provided only to facilitate descriptive interpretation and were not used to define the primary regression models or participant eligibility. Dashes indicate variables for which no single clinical cut-off was applied in this study. Smoking duration and number of cigarettes per day are reported for participants with available smoking data. Dyslipidemia was defined as previous clinical diagnosis, lipid-lowering treatment, or an abnormal lipid profile at assessment. Between-sex comparisons were performed using Student’s *t*-test with Welch correction when appropriate, the Mann–Whitney *U* test, the χ^2^ test, or Fisher’s exact test, as appropriate. Acute-phase hospitalization was available for 298 participants. Supplement-use information was available for 197 participants (57 men and 140 women); percentages for this variable were calculated using participants with available data as the denominator. Abbreviations: BMI, body mass index; DBP, diastolic blood pressure; FPG, fasting plasma glucose; GADS, Goldberg Anxiety and Depression Scale; HDL-c, high-density lipoprotein cholesterol; LDL-c, low-density lipoprotein cholesterol; MAP, mean arterial pressure; MD, Mediterranean diet; MEDAS, Mediterranean Diet Adherence Screener; MET, metabolic equivalent; SBP, systolic blood pressure.

**Table 2:** Cognitive performance, everyday cognitive complaints, and cognitive fatigue overall and by sex.

Variable	Overall (*n* = 304)	Men (*n* = 97)	Women (*n* = 207)	*p*-Value
MoCA total score	24.00 (21.00–26.00)	23.00 (21.00–25.00)	24.00 (21.00–26.00)	0.059
MIS	11.00 (9.00–13.00)	9.00 (7.00–11.00)	12.00 (10.00–13.00)	<0.001
MoCA normative z score	−0.32 (−0.82–0.19)	−0.45 (−0.79–0.15)	−0.29 (−0.86–0.21)	0.475
MIS normative z score	−0.32 (−0.76–0.14)	−0.32 (−0.76–0.16)	−0.35 (−0.74–0.11)	0.715
MoCA visuospatial/executive domain	4.00 (3.00–4.00)	4.00 (3.00–4.00)	4.00 (3.00–4.00)	0.707
MoCA naming domain	3.00 (3.00–3.00)	3.00 (3.00–3.00)	3.00 (3.00–3.00)	0.258
MoCA attention domain	5.00 (4.00–6.00)	5.00 (4.00–6.00)	5.00 (4.00–6.00)	0.423
MoCA language domain	2.00 (2.00–3.00)	2.00 (2.00–3.00)	2.00 (1.75–3.00)	0.752
MoCA abstraction domain	2.00 (1.00–2.00)	2.00 (1.00–2.00)	2.00 (1.00–2.00)	0.865
MoCA delayed recall domain	3.00 (2.00–4.00)	2.00 (1.00–3.00)	3.00 (2.00–4.00)	<0.001
MoCA orientation domain	6.00 (6.00–6.00)	6.00 (6.00–6.00)	6.00 (6.00–6.00)	0.995
MFE-30 total score	31.50 (16.00–53.00)	25.50 (14.00–42.00)	36.00 (18.25–55.00)	0.002
MFIS cognitive subscale	19.50 (7.00–29.00)	14.00 (4.00–26.00)	24.00 (11.00–31.00)	0.001

Values are presented as median (interquartile range). Valid observations were 299 for MoCA total score, MoCA normative z score, and MoCA domain scores; 297 for MIS and MIS normative z score; 298 for MFE-30 total score; and 296 for the MFIS cognitive subscale. Higher scores indicate better cognitive performance for MoCA, MIS, MoCA normative z scores, MIS normative z scores, and MoCA domain scores, whereas higher MFE-30 and MFIS cognitive scores indicate greater everyday cognitive complaints and cognitive fatigue, respectively. MoCA total score is reported without the standard education correction. MoCA and MIS scores were transformed into normative z scores using the Bayesian β-GAM normative approach described previously [45]. Between-sex comparisons were performed using the Mann–Whitney U test. MFE-30, Memory Failures of Everyday Questionnaire; MFIS, Modified Fatigue Impact Scale; MIS, Memory Index Score; MoCA, Montreal Cognitive Assessment.

**Table 3:** Dietary vitamin intake overall and by sex.

Variable	Overall (*n* = 304)	Men (*n* = 97)	Women (*n* = 207)	*p*-Value
Vitamin A, µg RE/day	893.14 [678.08–1076.60]	896.10 [677.65–1052.31]	890.60 [679.35–1094.03]	0.506
Vitamin B1, mg/day	1.41 [1.14–1.77]	1.40 [1.14–1.78]	1.41 [1.14–1.75]	0.904
Vitamin B2, mg/day	1.62 [1.29–1.96]	1.66 [1.24–1.98]	1.60 [1.31–1.96]	0.941
Vitamin B3, mg NE/day	33.16 [27.46–38.35]	34.11 [26.99–40.14]	32.55 [27.53–37.36]	0.384
Vitamin B6, mg/day	2.29 [1.81–2.83]	2.33 [1.75–2.69]	2.27 [1.86–2.86]	0.371
Vitamin B9, µg DFE/day	267.34 [217.42–334.46]	265.77 [224.43–343.96]	268.09 [215.66–331.35]	0.635
Vitamin B12, µg/day	8.03 [6.01–11.24]	7.49 [5.68–10.87]	8.20 [6.26–11.31]	0.154
Vitamin C, mg/day	141.60 [98.34–184.00]	137.39 [93.44–180.26]	143.39 [104.36–184.49]	0.505
Vitamin D, µg/day	5.34 [3.60–6.98]	5.25 [3.53–6.76]	5.45 [3.68–7.02]	0.403

Values are presented as median [interquartile range]. Between-sex comparisons were performed using the Mann–Whitney *U* test. Dietary adequacy is presented separately in Figure 2 as the percentage of participants meeting the sex-specific or common dietary reference values for each vitamin. Adequacy variables were used for descriptive purposes only, whereas regression models used vitamin intake as a continuous exposure. Vitamin intake estimates were derived from recorded foods and beverages and did not quantify vitamin intake from dietary supplements. Vitamin A was expressed as retinol equivalents, including provitamin A carotenoid contributions, and should not be interpreted as preformed retinol intake alone. DFE, dietary folate equivalents; NE, niacin equivalents; RE, retinol equivalents.

**Table 4:** Multivariable linear regression models of continuous vitamin intake and normative cognitive performance.

Model	Vitamin	MoCA Normative *z* Score, β (95% CI); Unadjusted *p*	MIS Normative *z* Score, β (95% CI); Unadjusted *p*
Model 1	Vitamin A, per 100 µg RE/day	−0.004 (−0.016 to 0.007); *p* = 0.478	−0.010 (−0.019 to 0.000); *p* = 0.054
Model 1	Vitamin B1, mg/day	0.038 (−0.190 to 0.267); *p* = 0.741	−0.153 (−0.406 to 0.101); *p* = 0.238
Model 1	Vitamin B2, mg/day	−0.001 (−0.186 to 0.183); *p* = 0.988	−0.044 (−0.230 to 0.142); *p* = 0.640
Model 1	Vitamin B3, mg/day	0.015 (−0.006 to 0.035); *p* = 0.157	0.007 (−0.018 to 0.032); *p* = 0.592
Model 1	Vitamin B6, mg/day	−0.066 (−0.203 to 0.072); *p* = 0.349	−0.150 (−0.310 to 0.010); *p* = 0.065
Model 1	Vitamin B9, µg/day	0.000 (−0.002 to 0.001); *p* = 0.787	−0.001 (−0.002 to 0.001); *p* = 0.485
Model 1	Vitamin B12, µg/day	−0.005 (−0.015 to 0.005); *p* = 0.313	−0.011 (−0.020 to −0.002); *p* = 0.014
Model 1	Vitamin C, mg/day	−0.001 (−0.003 to 0.000); *p* = 0.158	−0.002 (−0.004 to −0.001); *p* = 0.008
Model 1	Vitamin D, µg/day	0.011 (−0.021 to 0.043); *p* = 0.488	−0.006 (−0.037 to 0.026); *p* = 0.724
Model 2	Vitamin A, per 100 µg RE/day	−0.004 (−0.016 to 0.007); *p* = 0.469	−0.009 (−0.019 to 0.001); *p* = 0.082
Model 2	Vitamin B1, mg/day	0.002 (−0.231 to 0.235); *p* = 0.989	−0.184 (−0.451 to 0.084); *p* = 0.180
Model 2	Vitamin B2, mg/day	−0.029 (−0.217 to 0.159); *p* = 0.762	−0.059 (−0.262 to 0.143); *p* = 0.566
Model 2	Vitamin B3, mg/day	0.015 (−0.005 to 0.035); *p* = 0.151	0.006 (−0.019 to 0.031); *p* = 0.650
Model 2	Vitamin B6, mg/day	−0.086 (−0.227 to 0.056); *p* = 0.238	−0.164 (−0.330 to 0.002); *p* = 0.054
Model 2	Vitamin B9, µg/day	0.000 (−0.002 to 0.001); *p* = 0.823	−0.001 (−0.002 to 0.001); *p* = 0.513
Model 2	Vitamin B12, µg/day	−0.006 (−0.016 to 0.005); *p* = 0.298	−0.011 (−0.020 to −0.002); *p* = 0.015
Model 2	Vitamin C, mg/day	−0.001 (−0.003 to 0.001); *p* = 0.190	−0.002 (−0.004 to −0.001); *p* = 0.010
Model 2	Vitamin D, µg/day	0.007 (−0.024 to 0.038); *p* = 0.641	−0.010 (−0.042 to 0.021); *p* = 0.528

Values are unstandardized regression coefficients with 95% confidence intervals and unadjusted *p*-values. Model 1 was adjusted for total energy intake. Model 2 was adjusted for total energy intake, body mass index, and Mediterranean diet adherence. Each vitamin was entered as a continuous exposure in a separate model. For vitamin A, coefficients are expressed per 100 µg retinol equivalents/day to improve readability. Robust HC3 standard errors were used. Confidence intervals and p-values were calculated using the t distribution with the residual degrees of freedom of each model. Model-specific sample sizes were 286 for the MoCA normative *z*-score models and 284 for the MIS normative *z*-score models in both adjustment models. FDR-adjusted *q*-values are provided in Supplementary Table S5, and model diagnostics are reported in Supplementary Table S6. CI, confidence interval; FDR, false discovery rate; MIS, Memory Index Score; MoCA, Montreal Cognitive Assessment; RE, retinol equivalents.

## Data Availability

The data supporting the findings of this study are available on ZENODO at: https://doi.org/10.5281/zenodo.14282873.

## Supplementary Materials

The following supporting information can be downloaded at: https://www.mdpi.com/article/10.3390/nu18203370/s1. Table S1: STROBE checklist; Table S2: Published correlations between vitamin intake estimated using the EVIDENT application during the first week of recording and the food-frequency questionnaire; Table S3: Descriptive statistics for MoCA domain, item-level, and MoCA-derived scores; Table S4: Complete-case comparison for the primary fully adjusted normative cognitive models; Table S5: FDR-adjusted results for the primary normative cognitive models; Table S6: Diagnostic summary of the primary normative cognitive regression models and correlations among dietary vitamin intakes; Table S7: Vitamin B3 associations with the MoCA language domain and verbal fluency; Table S8: Sensitivity analyses of the vitamin B3 associations with the MoCA language domain and verbal fluency; Table S9: Multivariable models of continuous vitamin intake with everyday cognitive complaints and cognitive fatigue; Table S10: Sensitivity analyses using MoCA Memory and MoCA Multiple scores; Figure S1: Directed acyclic graph (DAG) informing covariate selection for analyses of vitamin B3 intake and cognitive performance; Figure S2: Adjusted association between vitamin B3 intake and verbal fluency.

## Abbreviations

The following abbreviations are used in this manuscript:

LC	Long COVID
APISAL	Primary Care Research Unit of Salamanca
WHO	World Health Organization
STROBE	Strengthening the Reporting of Observational Studies in Epidemiology
REDCap	Research Electronic Data Capture
IBSAL	Biomedical Research Institute of Salamanca
MoCA	Montreal Cognitive Assessment
MIS	Memory Index Score
MFE-30	Memory Failures of Everyday Questionnaire, 30-item version
MFIS	Modified Fatigue Impact Scale
GADS	Goldberg Anxiety and Depression Scale
MEDAS	Mediterranean Diet Adherence Screener
GPAQ	Global Physical Activity Questionnaire
BMI	Body mass index
FPG	Fasting plasma glucose
HDL-c	High-density lipoprotein cholesterol
LDL-c	Low-density lipoprotein cholesterol
RE	Retinol equivalents
DFE	Dietary folate equivalents
β-GAM	Bayesian beta generalized additive model
HC3	Heteroskedasticity-consistent covariance estimator type 3
OR	Odds ratio
SD	Standard deviation
DAG	Directed acyclic graph
FDR	False discovery rate
CI	Confidence interval
VIF	Variance inflation factor
