# Supplementary material for: Dietary Vitamin Intake and Cognitive Performance in Adults with Long COVID: The BioICOPER Study
Source: Nutrients. 2026 Oct 14;18(20):3370. doi: 10.3390/nu18203370 (PMC13708155; doi:10.3390/nu18203370)
Supplement: Supplementary file 1 [file nutrients-18-03370-s001.zip › nutrients-4588441-supplementary.pdf]

Table S1. STROBE Statement checklist of items.

Item No.	STROBE recommendation	Page No.	Relevant text / section from manuscript
1(a)	Indicate the study's design with a commonly used term in the title or the abstract.	1	Abstract, Methods: the study is explicitly described as a cross-sectional study ("A cross-sectional study was conducted in 304 adults with LC.").
1(b)	Provide in the abstract an informative and balanced summary of what was done and what was found.	1	Abstract: structured summary including background, objective, methods, results, and conclusions, reporting the absence of FDR-significant associations with global normative cognitive performance and the secondary vitamin B3–language association.
2	Explain the scientific background and rationale for the investigation being reported.	2–3	Section 1, Introduction: provides the scientific background on Long COVID and cognitive symptoms, summarizes the biological relevance of vitamins and the existing evidence on vitamins and cognition, and identifies the limited evidence regarding habitual dietary vitamin intake and cognitive performance in adults with Long COVID.
3	State specific objectives, including any prespecified hypotheses.	3	Section 1, Introduction: the final paragraph states the study objective of analyzing the relationship between dietary vitamin intake and cognitive performance in adults with Long COVID, considering both objective cognitive performance and self-reported cognition, and states the study

Item No.	STROBE recommendation	Page No.	Relevant text / section from manuscript
			hypothesis. The vitamin–cognition analysis was a secondary analysis of the BioICOPER cohort rather than a prospectively registered primary hypothesis.
4	Present key elements of study design early in the paper.	4	Section 2.1, Study Design and Participants: identifies the study as a cross-sectional observational analysis within the BioICOPER project and describes the study setting.
5	Describe the setting, locations, and relevant dates, including periods of recruitment, exposure, follow-up, and data collection.	4	Section 2.1, Study Design and Participants: describes the Primary Care Research Unit of Salamanca setting and the BioICOPER project context and reports recruitment from 9 March 2023 to 10 September 2024.
6	Give the eligibility criteria, and the sources and methods of selection of participants.	4	Section 2.1, Study Design and Participants: describes eligibility and participant selection, including diagnosis of Long COVID according to WHO criteria, consecutive recruitment, and the prespecified exclusion criteria.

Item No.	STROBE recommendation	Page No.	Relevant text / section from manuscript
7	Clearly define all outcomes, exposures, predictors, potential confounders, and effect modifiers. Give diagnostic criteria, if applicable.	5–6	Sections 2.3–2.6: define dietary vitamin intake as the exposure; objective and self-reported cognitive outcomes, including MoCA, MIS, MFE-30, and MFIS; sociodemographic, clinical, and lifestyle covariates; and the prespecified adjustment variables used in the multivariable and sensitivity analyses.
8	For each variable of interest, give sources of data and details of methods of assessment.	5–6	Sections 2.2–2.5: describe the sources of data and assessment methods for the main study variables, including standardized data collection and REDCap management, the seven-day EVIDENT dietary record, cognitive and affective instruments (MoCA, MIS, MFE-30, MFIS, and GADS), and sociodemographic, clinical, cardiometabolic, medication, lifestyle, and physical activity measures.
9	Describe any efforts to address potential sources of bias.	4–5, 7–8	Sections 2.1, 2.2, and 2.6: describe consecutive recruitment, standardized assessor training, independent data-quality review, complete-case comparisons, HC3 robust standard errors, model diagnostics, FDR correction, and sensitivity analyses to reduce potential sources of selection, measurement, and model-related bias.

Item No.	STROBE recommendation	Page No.	Relevant text / section from manuscript
10	Explain how the study size was arrived at.	4	Section 2.1, Study Design and Participants: describes the study as a secondary analysis of the established BioICOPER cohort and reports a post hoc minimum detectable-effect analysis. With approximately 80% power and $\alpha = 0.05$, the available sample could detect an incremental effect of approximately $f^2 = 0.028–0.029$, corresponding to a partial R^2 of approximately 0.027–0.028.
11	Explain how quantitative variables were handled in the analyses. If applicable, describe which groupings were chosen and why.	5, 7–8	Sections 2.3 and 2.6: describe dietary adequacy for descriptive purposes, continuous modeling of vitamin intake, transformation of MoCA and MIS scores into normative z scores, standardization of vitamin exposures per 1 SD for MoCA domain analyses, proportional-odds ordinal modeling of bounded MoCA domains, and logistic regression for binary item-level outcomes. Quasibinomial models were used as sensitivity analyses rather than as the primary domain-level model.
12(a)	Describe all statistical methods, including those used to control for confounding.	7–8	Section 2.6, Statistical Analysis: describes the statistical methods used for descriptive and multivariable analyses, including complete-case modeling, prespecified covariate adjustment, HC3 robust standard errors, proportional-odds ordinal regression for bounded MoCA domains,

Item No.	STROBE recommendation	Page No.	Relevant text / section from manuscript
			logistic regression for binary item-level outcomes, model diagnostics, multiple-imputation and other sensitivity analyses, and Benjamini–Hochberg FDR correction.
12(b)	Describe any methods used to examine subgroups and interactions.	7–8	Section 2.6, Statistical Analysis: describes the prespecified secondary and sensitivity analyses; no primary subgroup or interaction analyses were conducted.
12(c)	Explain how missing data were addressed.	7–8	Section 2.6, Statistical Analysis: describes examination of missing data by variable and by model, complete-case estimation of multivariable models, comparison of participants included in and excluded from the primary fully adjusted normative cognitive models, and multiple imputation as a sensitivity analysis.
12(d)	Cohort study: If applicable, explain how loss to follow-up was addressed	7–8	Section 2.6, Statistical Analysis: not applicable because this was a cross-sectional analysis and there was no longitudinal follow-up or loss to follow-up to address.

Item No.	STROBE recommendation	Page No.	Relevant text / section from manuscript
12(e)	Case-control study: If applicable, explain how matching of cases and controls was addressed.	7–8	Section 2.6, Statistical Analysis: not applicable because this was a cross-sectional analysis and no case–control matching was performed.
12(f)	Cross-sectional study: If applicable, describe analytical methods taking account of sampling strategy.	4, 7–8	Sections 2.1 and 2.6: participants were recruited consecutively, and the cross-sectional analyses used complete-case multivariable models with prespecified covariate adjustment and HC3 robust standard errors; no weighting or other sampling-design correction was required.
12(g)	Describe any sensitivity analyses.	7–8	Section 2.6, Statistical Analysis: describes sensitivity analyses with additional Long COVID–specific covariates, psychotropic medication use, protein intake, energy-reporting plausibility using the Goldberg approach, dietary supplement use, exclusion of supplement users, alternative quasibinomial and HC3 linear specifications for the B3–language association, multiple imputation, and models using MoCA Memory and MoCA Multiple scores.
13(a)	Report numbers of individuals at each stage of study.	4, Figure 1	Section 2.1, Study Design and Participants, and Figure 1: report the participant selection process, the number of participants assessed through the recruitment pathways, the final study sample of 304 adults with Long

Item No.	STROBE recommendation	Page No.	Relevant text / section from manuscript
			COVID, and the outcome-specific analytical sample sizes of n = 286 for normative MoCA and n = 284 for normative MIS.
13(b)	Give reasons for non-participation at each stage.	4, Figure 1	Figure 1 and Section 2.1, Study Design and Participants: describe the participant selection process and provide the reasons for non-inclusion at the different stages of recruitment.
13(c)	Consider use of a flow diagram.	4, Figure 1	Figure 1: presents the participant flow diagram, summarizing recruitment pathways, reasons for non-inclusion, the composition of the final study sample, and the analytical sample sizes for the primary global cognitive models.
14(a)	Give characteristics of study participants and information on exposures and potential confounders.	8–10	Section 3.1, Participant Characteristics at Study Inclusion, and Table 1: present sociodemographic, clinical, cardiometabolic, vaccination-related, and lifestyle characteristics of the study population overall and by sex, including variables used as potential confounders in the analyses.

Item No.	STROBE recommendation	Page No.	Relevant text / section from manuscript
14(b)	Indicate number of participants with missing data for each variable of interest.	11, 13; Supplementary Table S4	Table 2, Table 4, and Supplementary Table S4 report the number of valid observations for the main cognitive variables, model-specific complete-case sample sizes for the primary regression models, and the comparison between participants included in and excluded from the primary fully adjusted normative cognitive models.
15	Cross-sectional study: report numbers of outcome events or summary measures.	8, 11–13	Section 3, Results, and Tables 1–4: report summary measures for the study population, cognitive outcomes, dietary vitamin intake, and the primary regression estimates for normative cognitive performance.
16(a)	Give unadjusted estimates and, if applicable, confounder-adjusted estimates and their precision. Make clear which confounders were adjusted for and why they were included.	7–8, 13; Supplementary Tables S4–S10	Section 2.6, Statistical Analysis, Table 4, and **Supplementary Tables S4–S10** describe the covariate adjustment for Models 1 and 2 and report unstandardized regression coefficients with 95% confidence intervals and unadjusted p-values for the primary normative cognitive models; the supplementary tables provide FDR-adjusted results and additional secondary and sensitivity analyses.
16(b)	Report category boundaries when continuous variables were categorized.	5, 10–12, Table 1, Figure 2	Section 2.3, Dietary Intake Assessment, Table 1, and Figure 2: report the dietary reference values and clinical cut-offs used for descriptive

Item No.	STROBE recommendation	Page No.	Relevant text / section from manuscript
			categorization and interpretation. Dietary adequacy was used for descriptive purposes only, whereas the primary regression models analyzed vitamin intake as a continuous exposure.
16(c)	If relevant, consider translating estimates of relative risk into absolute risk for a meaningful time period.	Not applicable	Not applicable: the study analyzed continuous cognitive outcomes and did not estimate relative risks or other time-based risk measures requiring translation into absolute risk.
17	Report other analyses done, such as analyses of subgroups and interactions, and sensitivity analyses.	13–15, Supplementary Tables S6–S10	Sections 3.4–3.5 and Supplementary Tables S6–S10 report model diagnostics; proportional-odds ordinal analyses of MoCA domains and post hoc logistic analysis of verbal fluency; models for everyday cognitive complaints and cognitive fatigue; sensitivity analyses using MoCA Memory and MoCA Multiple scores; and additional sensitivity analyses of the vitamin B3–language and verbal-fluency findings, including Long COVID–specific covariates, protein intake, Goldberg energy-reporting plausibility, psychotropic medication, supplement use, and multiple imputation.

Item No.	STROBE recommendation	Page No.	Relevant text / section from manuscript
18	Summarize key results with reference to study objectives.	15–18	Section 4, Discussion: summarizes the key findings in relation to the study objective, emphasizing the absence of a general relationship between dietary vitamin intake and global cognitive performance, together with the secondary FDR-significant vitamin B3–language association and the post hoc exploratory verbal-fluency finding.
19	Discuss limitations of the study, taking into account sources of potential bias or imprecision. Discuss both direction and magnitude of any potential bias.	17	Section 4.1, Strengths and Limitations: discusses the main sources of potential bias and imprecision, including the cross-sectional design and potential reverse causation, dietary self-report and possible differential recording error, modest vitamin-specific correlations for EVIDENT, incomplete information on supplement use and inability to quantify vitamin exposure from supplements, inability to separate preformed niacin from niacin equivalents, absence of nutritional biomarkers, incomplete Long COVID–specific information, psychotropic medication, unmodeled seasonality, residual confounding, complete-case selection, inability to compare enrolled participants formally with persons who declined participation, restricted generalizability associated with

Item No.	STROBE recommendation	Page No.	Relevant text / section from manuscript
			exclusion of established cardiovascular disease, and limitations of MoCA as a screening instrument.
20	Give a cautious overall interpretation of results considering objectives, limitations, multiplicity of analyses, results from similar studies, and other relevant evidence.	15–18	Section 4, Discussion, and Section 4.1, Strengths and Limitations: provide a cautious interpretation of the findings in relation to the study objectives, the multiplicity-adjusted results, previous evidence on vitamins and cognition, the biological plausibility of the vitamin B3 finding, and the main methodological limitations and sensitivity analyses, while distinguishing the secondary B3–language association from the post hoc exploratory verbal-fluency analysis.
21	Discuss the generalisability of the study results.	17–18	Section 4.1, Strengths and Limitations, and Section 5, Conclusions: discuss the limits to generalisability arising from the cross-sectional design, dietary assessment, inability to compare formally with persons who declined participation, exclusion of individuals with established cardiovascular disease, use of Spanish normative data, brief cognitive screening, and the specific Long COVID clinical sample, and emphasize

Item No.	STROBE recommendation	Page No.	Relevant text / section from manuscript
			the need for replication in longitudinal studies with nutritional biomarkers and broader neuropsychological assessment.
22	Give the source of funding and the role of the funders.	19	Funding statement: identifies the sources of financial support and states that the funders had no role in the study design, data analysis, interpretation of the results, manuscript preparation, or the decision to submit the manuscript for publication.

Table S2. Published correlations between vitamin intake estimated using the EVIDENT application during the first week of recording and the food-frequency questionnaire.

Vitamin	Unit reported in the EVIDENT validation study	Correlation with FFQ, *r*	*p*-value
Vitamin A	μg/day	0.141	<0.01
Vitamin B1 (thiamine)	mg/day	0.155	<0.01
Vitamin B2 (riboflavin)	mg/day	0.205	<0.01
Vitamin B3 (niacin)	mg/day	0.130	<0.05
Vitamin B6	mg/day	0.210	<0.01
Vitamin B9 (folic acid)	μg/day	0.231	<0.01
Vitamin B12	μg/day	0.139	<0.01
Vitamin C	mg/day	0.262	<0.01
Vitamin D	μg/day	0.092	>0.05

Values are Pearson correlation coefficients between nutrient intake estimated from the first week of EVIDENT records and intake estimated using the food-frequency questionnaire [48]. Terminology and units follow the original publication. FFQ, food-frequency questionnaire.

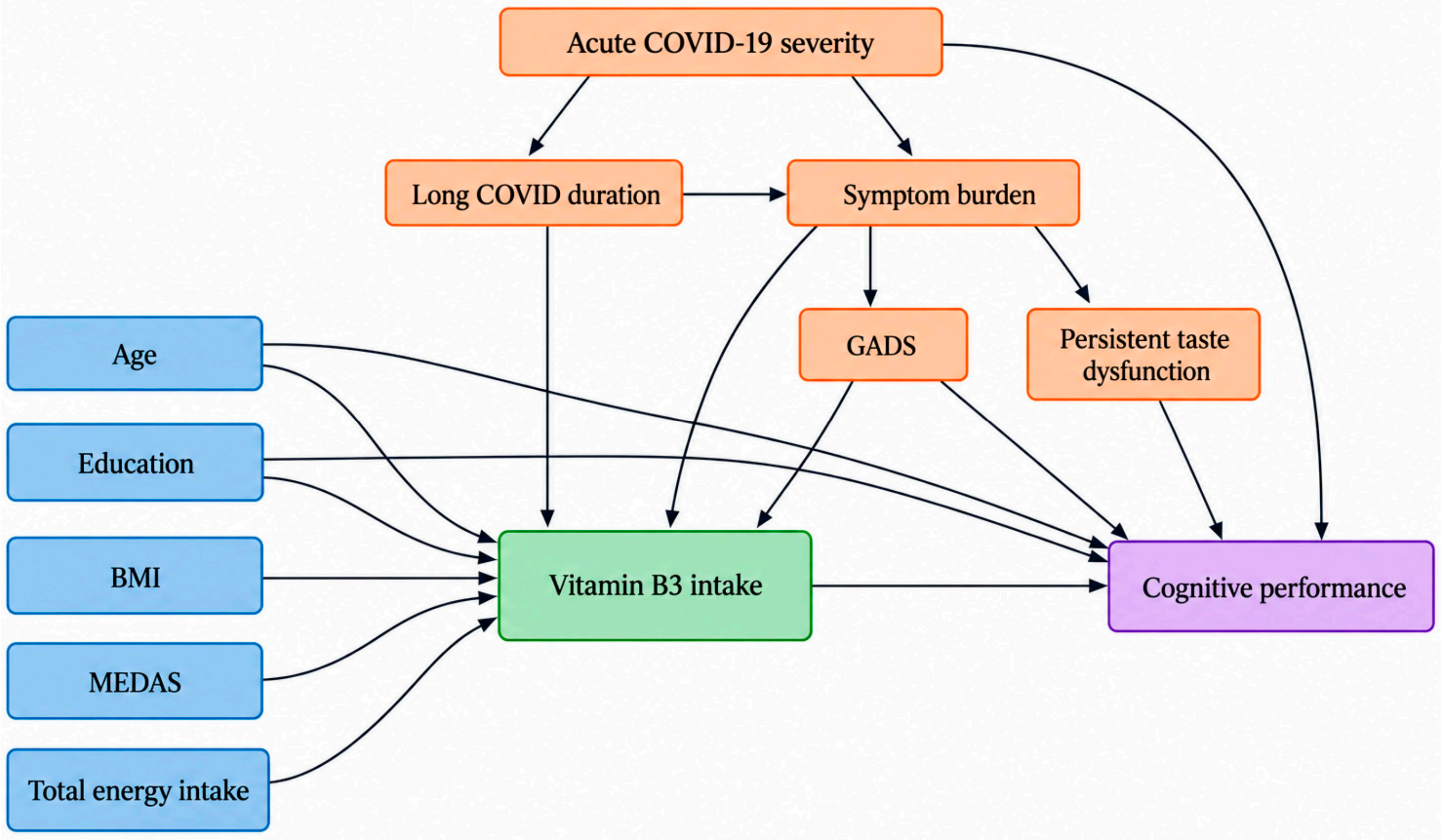

Figure S1. Directed acyclic graph (DAG) informing covariate selection for analyses of vitamin B3 intake and cognitive performance. The DAG represents the prespecified relationships among vitamin B3 intake, cognitive performance, demographic and nutritional covariates, and Long COVID–related clinical factors. Blue nodes denote demographic and nutritional covariates, orange nodes Long COVID–related clinical factors, the green node vitamin B3 intake, and the purple node cognitive performance. Arrows represent assumed directional relationships used to guide covariate selection and sensitivity analyses and should not be interpreted as empirically established causal effects. GADS, Goldberg Anxiety and Depression Scale; MEDAS, Mediterranean Diet Adherence Screener; BMI, body mass index.

Table S3. Descriptive statistics for MoCA domain, item-level, and MoCA-derived scores.

Domain / item	Score range	Mean ± SD	Median [IQR]	Floor, n (%)	Ceiling, n (%)	Skewness	Excess kurtosis
**Visuospatial/Executive**	0–5	3.48 ± 1.12	4.00 [3.00–4.00]	1 (0.3)	60 (20.1)	−0.43	−0.41
Alternation / trail-making	0–1	0.33 ± 0.47	0.00 [0.00–1.00]	201 (67.2)	98 (32.8)	0.74	−1.47
Cube copy	0–1	0.64 ± 0.48	1.00 [0.00–1.00]	108 (36.1)	191 (63.9)	−0.58	−1.67
Clock contour	0–1	0.98 ± 0.14	1.00 [1.00–1.00]	6 (2.0)	293 (98.0)	−6.88	45.63
Clock numbers	0–1	0.80 ± 0.40	1.00 [1.00–1.00]	60 (20.1)	239 (79.9)	−1.50	0.26
Clock hands	0–1	0.74 ± 0.44	1.00 [0.00–1.00]	79 (26.4)	220 (73.6)	−1.07	−0.85
**Naming**	0–3	2.90 ± 0.32	3.00 [3.00–3.00]	0 (0.0)	272 (91.0)	−3.35	11.28
Naming: lion	0–1	1.00 ± 0.00	1.00 [1.00–1.00]	0 (0.0)	299 (100.0)	0.00	0.00
Naming: rhinoceros	0–1	0.91 ± 0.28	1.00 [1.00–1.00]	26 (8.7)	273 (91.3)	−2.95	6.73
Naming: camel	0–1	0.99 ± 0.10	1.00 [1.00–1.00]	3 (1.0)	296 (99.0)	−9.88	96.30
**Attention**	0–6	4.80 ± 1.23	5.00 [4.00–6.00]	2 (0.7)	107 (35.8)	−1.09	1.11
Digit span forward	0–1	0.77 ± 0.42	1.00 [1.00–1.00]	70 (23.4)	229 (76.6)	−1.26	−0.41
Digit span backward	0–1	0.76 ± 0.43	1.00 [1.00–1.00]	72 (24.1)	227 (75.9)	−1.22	−0.52
Vigilance	0–1	0.86 ± 0.35	1.00 [1.00–1.00]	42 (14.0)	257 (86.0)	−2.08	2.34
Serial subtraction	0–3	2.41 ± 0.86	3.00 [2.00–3.00]	16 (5.4)	182 (60.9)	−1.42	1.17

Domain / item	Score range	Mean ± SD	Median [IQR]	Floor, n (%)	Ceiling, n (%)	Skewness	Excess kurtosis
**Language**	0–3	2.06 ± 0.85	2.00 [2.00–3.00]	13 (4.3)	104 (34.8)	−0.56	−0.43
Sentence repetition 1	0–1	0.90 ± 0.31	1.00 [1.00–1.00]	31 (10.4)	268 (89.6)	−2.61	4.86
Sentence repetition 2	0–1	0.62 ± 0.49	1.00 [0.00–1.00]	114 (38.1)	185 (61.9)	−0.49	−1.77
Verbal fluency	0–1	0.55 ± 0.50	1.00 [0.00–1.00]	135 (45.2)	164 (54.8)	−0.20	−1.97
**Abstraction**	0–2	1.54 ± 0.59	2.00 [1.00–2.00]	14 (4.7)	175 (58.5)	−0.86	−0.25
Train–bicycle similarity	0–1	0.94 ± 0.24	1.00 [1.00–1.00]	19 (6.4)	280 (93.6)	−3.60	11.01
Watch–ruler similarity	0–1	0.60 ± 0.49	1.00 [0.00–1.00]	119 (39.8)	180 (60.2)	−0.42	−1.84
**Delayed recall**	0–5	2.59 ± 1.52	3.00 [2.00–4.00]	39 (13.0)	36 (12.0)	−0.15	−0.87
Delayed recall: face	0–1	0.48 ± 0.50	0.00 [0.00–1.00]	154 (51.5)	145 (48.5)	0.06	−2.01
Delayed recall: silk	0–1	0.49 ± 0.50	0.00 [0.00–1.00]	151 (50.5)	148 (49.5)	0.02	−2.01
Delayed recall: temple	0–1	0.47 ± 0.50	0.00 [0.00–1.00]	158 (52.8)	141 (47.2)	0.11	−2.00
Delayed recall: carnation	0–1	0.45 ± 0.50	0.00 [0.00–1.00]	165 (55.2)	134 (44.8)	0.21	−1.97
Delayed recall: red	0–1	0.69 ± 0.47	1.00 [0.00–1.00]	94 (31.4)	205 (68.6)	−0.80	−1.36
**Orientation**	0–6	5.91 ± 0.36	6.00 [6.00–6.00]	0 (0.0)	277 (92.6)	−5.10	31.67
Date	0–1	0.96 ± 0.20	1.00 [1.00–1.00]	12 (4.0)	287 (96.0)	−4.71	20.32
Month	0–1	0.99 ± 0.08	1.00 [1.00–1.00]	2 (0.7)	297 (99.3)	−12.17	146.97

Domain / item	Score range	Mean ± SD	Median [IQR]	Floor, n (%)	Ceiling, n (%)	Skewness	Excess kurtosis
Year	0–1	0.99 ± 0.08	1.00 [1.00–1.00]	2 (0.7)	297 (99.3)	−12.17	146.97
Day	0–1	0.99 ± 0.10	1.00 [1.00–1.00]	3 (1.0)	296 (99.0)	−9.88	96.30
Place	0–1	0.98 ± 0.15	1.00 [1.00–1.00]	7 (2.3)	292 (97.7)	−6.34	38.40
City	0–1	1.00 ± 0.06	1.00 [1.00–1.00]	1 (0.3)	298 (99.7)	−17.29	299.00
**MoCA-derived scores**							
MoCA Memory score	0–5	2.59 ± 1.52	3.00 [2.00–4.00]	39 (13.04)	36 (12.04)	−0.15	−0.87
MoCA Multiple score	0–9	6.97 ± 1.54	7.00 [6.00–8.00]	0 (0.00)	44 (14.7)	−0.86	0.69

Values are based on 299 participants with a valid uncorrected MoCA score; five participants without valid MoCA data were excluded from these summaries. Domain scores were calculated as strict sums of their component item scores, with items displayed immediately below the corresponding domain. MoCA-derived scores are presented separately at the end of the table. MoCA Memory and MoCA Multiple scores correspond to raw sum scores from the item sets defining the 12-item two-factor MoCA solution [44] and were used in sensitivity analyses. They should not be interpreted as standardized latent factor scores. Mean ± standard deviation and median [interquartile range] are both reported because MoCA domain, item-level, and derived scores are discrete and bounded. Floor and ceiling effects were defined as the proportion of valid observations at the theoretical minimum and maximum score, respectively. Skewness and excess kurtosis were estimated using moment-based estimators. MoCA, Montreal Cognitive Assessment.

Table S4. Complete-case comparison for the primary fully adjusted normative cognitive models.

Panel	Variable	Included	Excluded	*p*-value
**A. MoCA normative models**		***n* = 286**	***n* = 18**	
	Age, years	53.35 ± 11.70	43.67 ± 11.82	0.003
	Female sex, n (%)	195 (68.2)	12 (66.7)	0.894
	Educational level, n (%)			0.168
	Primary education	33 (11.5)	0 (0.0)	
	Secondary education	146 (51.0)	8 (44.4)	
	University education	107 (37.4)	10 (55.6)	
	MoCA score	24.00 [21.00–26.00]	23.00 [23.00–25.00]	0.958
	MIS score	11.00 [9.00–13.00]	10.00 [9.00–12.00]	0.812
	MoCA normative *z* score	−0.32 [−0.79–0.21]	−0.63 [−1.10–−0.24]	0.168
	MIS normative *z* score	−0.32 [−0.76–0.14]	−0.43 [−0.97–−0.27]	0.236
	MFE-30 total score	31.00 [16.00–53.00]	32.00 [15.00–54.00]	0.680
	MFIS cognitive subscale	20.00 [7.00–29.00]	19.00 [4.00–32.00]	0.801
	Energy intake, kcal/day	1732.11 [1394.83–2043.38]	1532.54 [1328.18–1721.70]	0.273
	Vitamin A, μg RE/day	895.89 [678.08–1079.83]	766.90 [726.30–842.20]	0.608

Panel	Variable	Included	Excluded	*p*-value
	Vitamin B1, mg/day	1.41 [1.14–1.77]	1.16 [1.09–1.30]	0.245
	Vitamin B2, mg/day	1.62 [1.29–1.97]	1.37 [1.35–1.43]	0.223
	Vitamin B3, mg/day	33.18 [27.46–38.61]	31.24 [28.73–32.82]	0.507
	Vitamin B6, mg/day	2.31 [1.82–2.83]	1.75 [1.49–2.20]	0.211
	Vitamin B9, µg/day	270.62 [217.42–334.46]	246.80 [234.32–261.52]	0.458
	Vitamin B12, µg/day	8.11 [6.02–11.30]	6.05 [5.17–6.75]	0.081
	Vitamin C, mg/day	141.60 [98.34–184.41]	136.92 [94.97–172.30]	0.699
	Vitamin D, µg/day	5.34 [3.59–7.00]	5.46 [5.01–5.90]	0.850
	BMI, kg/m^2	27.77 [23.72–31.24]	24.94 [21.47–27.88]	0.053
	Mediterranean diet adherence	8.00 [7.00–9.00]	8.00 [6.00–9.75]	0.945
**B. MIS normative models**		***n* = 284**	***n* = 20**	
	Age, years	53.28 ± 11.71	45.60 ± 12.67	0.015
	Female sex, n (%)	194 (68.3)	13 (65.0)	0.759
	Educational level, n (%)			0.218
	Primary education	33 (11.6)	0 (0.0)	
	Secondary education	144 (50.7)	10 (50.0)	

Panel	Variable	Included	Excluded	*p*-value
	University education	107 (37.7)	10 (50.0)	
	MoCA score	24.00 [21.00–26.00]	23.00 [22.00–25.00]	0.661
	MIS score	11.00 [9.00–13.00]	10.00 [9.00–12.00]	0.812
	MoCA normative *z* score	−0.32 [−0.80–0.21]	−0.57 [−1.06–−0.27]	0.175
	MIS normative *z* score	−0.32 [−0.76–0.14]	−0.43 [−0.97–−0.27]	0.236
	MFE-30 total score	31.00 [16.00–52.50]	32.00 [16.50–53.50]	0.754
	MFIS cognitive subscale	20.00 [7.00–29.00]	14.00 [5.00–31.00]	0.550
	Energy intake, kcal/day	1732.11 [1392.97–2046.40]	1707.78 [1453.22–1791.04]	0.508
	Vitamin A, µg RE/day	895.89 [677.57–1081.48]	766.90 [727.56–945.06]	0.642
	Vitamin B1, mg/day	1.41 [1.14–1.77]	1.28 [1.15–1.45]	0.367
	Vitamin B2, mg/day	1.62 [1.29–1.97]	1.47 [1.36–1.73]	0.775
	Vitamin B3, mg/day	33.18 [27.44–38.51]	31.98 [31.16–35.92]	0.854
	Vitamin B6, mg/day	2.31 [1.81–2.83]	2.05 [1.62–2.52]	0.367
	Vitamin B9, µg/day	270.80 [216.90–334.84]	260.04 [241.07–263.48]	0.477
	Vitamin B12, µg/day	8.13 [6.03–11.33]	6.05 [5.07–6.91]	0.040
	Vitamin C, mg/day	141.88 [98.01–185.18]	119.09 [100.23–159.81]	0.471

Panel	Variable	Included	Excluded	*p*-value
	Vitamin D, μg/day	5.38 [3.59–7.00]	4.98 [4.91–5.67]	0.800
	BMI, kg/m²	27.74 [23.71–31.24]	26.08 [21.55–30.53]	0.220
	Mediterranean diet adherence	8.00 [7.00–9.00]	8.00 [6.00–10.00]	0.969

Complete cases were defined separately for each primary normative cognitive outcome according to the variables required for the fully adjusted model family, including the corresponding normative z score, all prespecified vitamin intake variables, total energy intake, body mass index, and Mediterranean diet adherence. The MoCA normative analyses included 286 participants and excluded 18 because of incomplete data; the MIS normative analyses included 284 participants and excluded 20. Age, sex, educational level, raw cognitive scores, MFE-30, and MFIS cognitive scores are presented for comparison but did not define complete-case status. Within the excluded groups, descriptive statistics and between-group comparisons were based on the available observations for each variable; therefore, the number of observations may vary across rows. Continuous variables are presented as mean ± standard deviation or median [interquartile range], according to their distribution. Comparisons were performed using Welch's t-test or the Mann–Whitney U test for continuous variables and the χ^2 test or Fisher's exact test for categorical variables, as appropriate. Vitamin B3 refers to niacin-equivalent intake. BMI, body mass index; MFE-30, Memory Failures of Everyday Questionnaire; MFIS, Modified Fatigue Impact Scale; MIS, Memory Index Score; MoCA, Montreal Cognitive Assessment; RE, retinol equivalents.

Table S5. FDR-adjusted results for the primary normative cognitive models.

Outcome	Model	Vitamin	β (95% CI); unadjusted *p*	FDR *q*-value
MoCA normative *z* score	Model 1	Vitamin A, per 100 μg RE/day	−0.004 (−0.016 to 0.007); *p* = 0.478	0.793
MoCA normative *z* score	Model 1	Vitamin B1, mg/day	0.038 (−0.190 to 0.267); *p* = 0.741	0.857
MoCA normative *z* score	Model 1	Vitamin B2, mg/day	−0.001 (−0.186 to 0.183); *p* = 0.988	0.989
MoCA normative *z* score	Model 1	Vitamin B3, mg/day	0.015 (−0.006 to 0.035); *p* = 0.157	0.517
MoCA normative *z* score	Model 1	Vitamin B6, mg/day	−0.066 (−0.203 to 0.072); *p* = 0.349	0.698
MoCA normative *z* score	Model 1	Vitamin B9, μg/day	0.000 (−0.002 to 0.001); *p* = 0.787	0.859
MoCA normative *z* score	Model 1	Vitamin B12, μg/day	−0.005 (−0.015 to 0.005); *p* = 0.313	0.664
MoCA normative *z* score	Model 1	Vitamin C, mg/day	−0.001 (−0.003 to 0.000); *p* = 0.158	0.517
MoCA normative *z* score	Model 1	Vitamin D, μg/day	0.011 (−0.021 to 0.043); *p* = 0.488	0.793
MoCA normative *z* score	Model 2	Vitamin A, per 100 μg RE/day	−0.004 (−0.016 to 0.007); *p* = 0.469	0.793
MoCA normative *z* score	Model 2	Vitamin B1, mg/day	0.002 (−0.231 to 0.235); *p* = 0.989	0.989
MoCA normative *z* score	Model 2	Vitamin B2, mg/day	−0.029 (−0.217 to 0.159); *p* = 0.762	0.857
MoCA normative *z* score	Model 2	Vitamin B3, mg/day	0.015 (−0.005 to 0.035); *p* = 0.151	0.517
MoCA normative *z* score	Model 2	Vitamin B6, mg/day	−0.086 (−0.227 to 0.056); *p* = 0.238	0.571
MoCA normative *z* score	Model 2	Vitamin B9, μg/day	0.000 (−0.002 to 0.001); *p* = 0.823	0.872

Outcome	Model	Vitamin	β (95% CI); unadjusted *p*	FDR *q*-value
MoCA normative *z* score	Model 2	Vitamin B12, μg/day	−0.006 (−0.016 to 0.005); *p* = 0.298	0.664
MoCA normative *z* score	Model 2	Vitamin C, mg/day	−0.001 (−0.003 to 0.001); *p* = 0.190	0.526
MoCA normative *z* score	Model 2	Vitamin D, μg/day	0.007 (−0.024 to 0.038); *p* = 0.641	0.807
MIS normative *z* score	Model 1	Vitamin A, per 100 μg RE/day	−0.010 (−0.019 to 0.000); *p* = 0.054	0.325
MIS normative *z* score	Model 1	Vitamin B1, mg/day	−0.153 (−0.406 to 0.101); *p* = 0.238	0.571
MIS normative *z* score	Model 1	Vitamin B2, mg/day	−0.044 (−0.230 to 0.142); *p* = 0.640	0.807
MIS normative *z* score	Model 1	Vitamin B3, mg/day	0.007 (−0.018 to 0.032); *p* = 0.592	0.807
MIS normative *z* score	Model 1	Vitamin B6, mg/day	−0.150 (−0.310 to 0.010); *p* = 0.065	0.337
MIS normative *z* score	Model 1	Vitamin B9, μg/day	−0.001 (−0.002 to 0.001); *p* = 0.485	0.793
MIS normative *z* score	Model 1	Vitamin B12, μg/day	−0.011 (−0.020 to −0.002); *p* = 0.014	0.134
MIS normative *z* score	Model 1	Vitamin C, mg/day	−0.002 (−0.004 to −0.001); *p* = 0.008	0.134
MIS normative *z* score	Model 1	Vitamin D, μg/day	−0.006 (−0.037 to 0.026); *p* = 0.724	0.857
MIS normative *z* score	Model 2	Vitamin A, per 100 μg RE/day	−0.009 (−0.019 to 0.001); *p* = 0.082	0.367
MIS normative *z* score	Model 2	Vitamin B1, mg/day	−0.184 (−0.451 to 0.084); *p* = 0.180	0.526
MIS normative *z* score	Model 2	Vitamin B2, mg/day	−0.059 (−0.262 to 0.143); *p* = 0.566	0.807
MIS normative *z* score	Model 2	Vitamin B3, mg/day	0.006 (−0.019 to 0.031); *p* = 0.650	0.807

Outcome	Model	Vitamin	β (95% CI); unadjusted *p*	FDR *q*-value
MIS normative *z* score	Model 2	Vitamin B6, mg/day	−0.164 (−0.330 to 0.002); $p = 0.054$	0.325
MIS normative *z* score	Model 2	Vitamin B9, μg/day	−0.001 (−0.002 to 0.001); $p = 0.513$	0.793
MIS normative *z* score	Model 2	Vitamin B12, μg/day	−0.011 (−0.020 to −0.002); $p = 0.015$	0.134
MIS normative *z* score	Model 2	Vitamin C, mg/day	−0.002 (−0.004 to −0.001); $p = 0.010$	0.134
MIS normative *z* score	Model 2	Vitamin D, μg/day	−0.010 (−0.042 to 0.021); $p = 0.528$	0.793

FDR-adjusted *q*-values were calculated using the Benjamini–Hochberg procedure across the 36 primary vitamin–outcome–model contrasts. Adjusted *q*-values were calculated from the full-precision unadjusted *p*-values before rounding for presentation. Model-specific sample sizes were 286 for MoCA normative z-score models and 284 for MIS normative z-score models in both adjustment models. Values are unstandardized regression coefficients with 95% confidence intervals and unadjusted *p*-values. Models used HC3 robust standard errors; confidence intervals and p-values were calculated using the t distribution with the residual degrees of freedom of each model. Model 1 was adjusted for total energy intake. Model 2 was adjusted for total energy intake, body mass index, and Mediterranean diet adherence. Each vitamin was entered as a continuous exposure in a separate model. For vitamin A, coefficients are expressed per 100 μg retinol equivalents/day. CI, confidence interval; FDR, false discovery rate; MIS, Memory Index Score; MoCA, Montreal Cognitive Assessment; RE, retinol equivalents.

Table S6. Diagnostic summary of the primary normative cognitive regression models and correlations among dietary vitamin intakes.

Panel A. Regression model diagnostics

Outcome	Model	Vitamin	R^2	Adj. R^2	Max VIF	Max |rstudent|	Max leverage	Max Cook's D
MoCA normative *z* score	Model 1	Vitamin A	0.008	0.001	1.05	3.16	0.282	0.195
MoCA normative *z* score	Model 1	Vitamin B1	0.007	0.000	2.00	3.18	0.307	0.046
MoCA normative *z* score	Model 1	Vitamin B2	0.007	0.000	1.74	3.16	0.181	0.041
MoCA normative *z* score	Model 1	Vitamin B3	0.014	0.007	3.00	3.22	0.091	0.062
MoCA normative *z* score	Model 1	Vitamin B6	0.009	0.002	1.57	3.18	0.101	0.058
MoCA normative *z* score	Model 1	Vitamin B9	0.007	0.000	1.76	3.16	0.107	0.110
MoCA normative *z* score	Model 1	Vitamin B12	0.011	0.004	1.03	3.15	0.184	0.126
MoCA normative *z* score	Model 1	Vitamin C	0.014	0.007	1.22	3.11	0.091	0.088
MoCA normative *z* score	Model 1	Vitamin D	0.008	0.001	1.14	3.17	0.317	0.047
MoCA normative *z* score	Model 2	Vitamin A	0.034	0.020	1.05	3.20	0.285	0.120
MoCA normative *z* score	Model 2	Vitamin B1	0.033	0.019	2.03	3.20	0.311	0.034
MoCA normative *z* score	Model 2	Vitamin B2	0.033	0.019	1.77	3.20	0.183	0.039
MoCA normative *z* score	Model 2	Vitamin B3	0.039	0.026	3.01	3.26	0.093	0.039
MoCA normative *z* score	Model 2	Vitamin B6	0.037	0.023	1.60	3.17	0.102	0.052

Panel A. Regression model diagnostics

Outcome	Model	Vitamin	R^2	Adj. R^2	Max VIF	Max |rstudent|	Max leverage	Max Cook's D
MoCA normative *z* score	Model 2	Vitamin B9	0.033	0.019	1.77	3.20	0.128	0.062
MoCA normative *z* score	Model 2	Vitamin B12	0.037	0.023	1.04	3.19	0.185	0.107
MoCA normative *z* score	Model 2	Vitamin C	0.039	0.025	1.22	3.15	0.092	0.053
MoCA normative *z* score	Model 2	Vitamin D	0.033	0.019	1.15	3.20	0.326	0.043
MIS normative *z* score	Model 1	Vitamin A	0.010	0.003	1.05	2.54	0.282	0.123
MIS normative *z* score	Model 1	Vitamin B1	0.008	0.001	2.00	2.59	0.308	0.130
MIS normative *z* score	Model 1	Vitamin B2	0.005	−0.002	1.75	2.54	0.182	0.124
MIS normative *z* score	Model 1	Vitamin B3	0.006	−0.002	2.99	2.53	0.091	0.135
MIS normative *z* score	Model 1	Vitamin B6	0.014	0.007	1.57	2.68	0.101	0.125
MIS normative *z* score	Model 1	Vitamin B9	0.006	−0.001	1.77	2.57	0.107	0.176
MIS normative *z* score	Model 1	Vitamin B12	0.017	0.010	1.03	2.59	0.185	0.119
MIS normative *z* score	Model 1	Vitamin C	0.024	0.017	1.22	2.64	0.092	0.119
MIS normative *z* score	Model 1	Vitamin D	0.005	−0.002	1.14	2.53	0.318	0.134
MIS normative *z* score	Model 2	Vitamin A	0.040	0.026	1.05	2.98	0.285	0.074
MIS normative *z* score	Model 2	Vitamin B1	0.040	0.026	2.03	2.98	0.311	0.077

Panel A. Regression model diagnostics

Outcome	Model	Vitamin	R^2	Adj. R^2	Max VIF	Max |rstudent|	Max leverage	Max Cook's D
MIS normative *z* score	Model 2	Vitamin B2	0.036	0.022	1.78	3.01	0.184	0.075
MIS normative *z* score	Model 2	Vitamin B3	0.036	0.022	3.01	2.94	0.093	0.081
MIS normative *z* score	Model 2	Vitamin B6	0.046	0.032	1.59	3.04	0.102	0.073
MIS normative *z* score	Model 2	Vitamin B9	0.037	0.023	1.77	2.97	0.129	0.107
MIS normative *z* score	Model 2	Vitamin B12	0.048	0.035	1.04	3.00	0.185	0.071
MIS normative *z* score	Model 2	Vitamin C	0.053	0.039	1.22	3.09	0.093	0.073
MIS normative *z* score	Model 2	Vitamin D	0.036	0.022	1.15	3.02	0.326	0.079

Panel B. Spearman correlation matrix of dietary vitamin intakes

Vitamin	A	B1	B2	B3	B6	B9	B12	C	D
A	1.000	0.419	0.532	0.460	0.554	0.625	0.389	0.517	0.314
B1	0.419	1.000	0.782	0.738	0.655	0.564	0.445	0.461	0.404
B2	0.532	0.782	1.000	0.702	0.701	0.570	0.481	0.414	0.433
B3	0.460	0.738	0.702	1.000	0.720	0.588	0.517	0.382	0.516
B6	0.554	0.655	0.701	0.720	1.000	0.555	0.575	0.451	0.429
B9	0.625	0.564	0.570	0.588	0.555	1.000	0.233	0.721	0.341

Vitamin	A	B1	B2	B3	B6	B9	B12	C	D
B12	0.389	0.445	0.481	0.517	0.575	0.233	1.000	0.140	0.502
C	0.517	0.461	0.414	0.382	0.451	0.721	0.140	1.000	0.169
D	0.314	0.404	0.433	0.516	0.429	0.341	0.502	0.169	1.000

Diagnostics in **Panel A** correspond to the primary multivariable linear regression models reported in Table 4. Model-specific sample sizes were 286 for MoCA normative z-score models and 284 for MIS normative z-score models in both adjustment models. Maximum VIF denotes the largest variance inflation factor observed in each model. Maximum leverage, maximum absolute studentized residual, and maximum Cook's distance are reported as influence diagnostics. Model 1 was adjusted for total energy intake. Model 2 was adjusted for total energy intake, body mass index, and Mediterranean diet adherence. **Panel B** presents unadjusted Spearman correlation coefficients calculated among 290 participants with complete data for all nine vitamin intakes. MIS, Memory Index Score; MoCA, Montreal Cognitive Assessment; VIF, variance inflation factor.

Table S7. Vitamin B3 associations with the MoCA language domain and verbal fluency.

Outcome	Model/specification	Exposure scale	Effect estimate (95% CI)	Unadjusted *p*-value	FDR-adjusted *q*-value
MoCA language domain	Proportional-odds ordinal, Model 1	Per 1 SD	OR = 2.19 (1.46–3.28)	<0.001	0.0116
MoCA language domain	Proportional-odds ordinal, Model 2	Per 1 SD	OR = 2.18 (1.45–3.27)	<0.001	0.0116
Verbal fluency	Logistic regression, Model 2	Per 5 mg/day	OR = 1.69 (1.30–2.19)	<0.001	—

Language-domain estimates were obtained using proportional-odds ordinal regression and are expressed as odds ratios per 1-SD increase in vitamin B3 intake (SD = 9.14 mg/day). FDR-adjusted *q*-values for the language-domain models were calculated using the Benjamini–Hochberg procedure across the prespecified family of 126 domain-level contrasts. Both ordinal models included 286 participants. Verbal fluency was examined post hoc using logistic regression in 286 participants and is reported per 5-mg/day increase in vitamin B3 intake. Because the verbal-fluency analysis was exploratory and was not included in the prespecified domain-level multiplicity family, an FDR-adjusted *q*-value was not calculated for this analysis. Model 1 was adjusted for age, sex, educational level, and total energy intake; Model 2 additionally included body mass index and Mediterranean diet adherence. Vitamin B3 refers to niacin-equivalent intake. CI, confidence interval; FDR, false discovery rate; MoCA, Montreal Cognitive Assessment; OR, odds ratio; SD, standard deviation.

Table S8. Sensitivity analyses of the vitamin B3 associations with the MoCA language domain and verbal fluency.

Sensitivity analysis	Outcome	Exposure scale	Effect estimate (95% CI)	Unadjusted *p*-value
Quasibinomial, Model 1	MoCA language domain	Per 1 mg/day	OR = 1.06 (1.03–1.09)	<0.001
Quasibinomial, Model 2	MoCA language domain	Per 1 mg/day	OR = 1.06 (1.03–1.09)	<0.001
Linear HC3, Model 1	MoCA language domain	Per 1 mg/day	β = 0.0347 (0.0161–0.0533)	<0.001
Linear HC3, Model 2	MoCA language domain	Per 1 mg/day	β = 0.0342 (0.0155–0.0529)	<0.001
Additional adjustment for protein intake	MoCA language domain	Per 1 mg/day	OR = 1.06 (1.03–1.09)	<0.001
Additional adjustment for protein intake	Verbal fluency	Per 5 mg/day	OR = 1.70 (1.30–2.22)	<0.001
Goldberg plausible energy reporters	MoCA language domain	Per 1 mg/day	OR = 1.07 (1.03–1.11)	<0.001
Goldberg plausible energy reporters	Verbal fluency	Per 5 mg/day	OR = 1.76 (1.28–2.43)	<0.001
Long COVID core adjustment	MoCA language domain	Per 1 SD	OR = 2.24 (1.53–3.28)	<0.001
Extended Long COVID adjustment	MoCA language domain	Per 1 SD	OR = 2.31 (1.56–3.41)	<0.001
Additional adjustment for any psychotropic medication	MoCA language domain	Per 1 mg/day	OR = 1.06 (1.03–1.09)	<0.001

Sensitivity analysis	Outcome	Exposure scale	Effect estimate (95% CI)	Unadjusted *p*-value
Additional adjustment for any psychotropic medication	Verbal fluency	Per 5 mg/day	OR = 1.72 (1.32–2.25)	<0.001
Additional adjustment for supplement use	MoCA language domain	Per 1 mg/day	OR = 1.07 (1.03–1.11)	0.001
Additional adjustment for supplement use	Verbal fluency	Per 5 mg/day	OR = 2.05 (1.44–2.93)	<0.001
Exclusion of supplement users	MoCA language domain	Per 1 mg/day	OR = 1.06 (1.02–1.10)	0.002
Exclusion of supplement users	Verbal fluency	Per 5 mg/day	OR = 1.97 (1.38–2.81)	<0.001
Multiple imputation	MoCA language domain	Per 1 mg/day	OR = 1.059 (1.028–1.092)	<0.001
Multiple imputation	Verbal fluency	Per 1 mg/day	OR = 1.111 (1.054–1.170)	<0.001

Sensitivity analyses evaluated the robustness of the vitamin B3–language association and the exploratory vitamin B3–verbal-fluency association under alternative model specifications and additional adjustment, restriction, and missing-data approaches. Quasibinomial and linear HC3 models included 286 participants. Analyses additionally adjusted for protein intake included 279 participants, and analyses restricted to plausible energy reporters included 164 participants. Long COVID–specific ordinal models included 280 participants. Psychotropic-medication sensitivity analyses included 286 participants. Supplement-adjusted analyses included 183 participants, whereas analyses excluding supplement users included 174 participants. Multiple-imputation estimates were pooled across 20 imputed datasets. The multiple-imputation results presented in this table refer specifically to the vitamin B3–language and vitamin B3–verbal-fluency sensitivity analyses and do not represent the full family of primary global cognitive models. Language-domain estimates from the Long COVID–specific models are expressed per 1-SD increase in vitamin B3 intake; other language-domain sensitivity estimates are expressed per 1-mg/day increase. Verbal-fluency estimates are expressed per 5-mg/day increase except for the multiple-imputation estimate, which is reported per 1-mg/day increase. Sensitivity analyses were not used for the primary multiplicity-adjusted significance decision and therefore FDR-adjusted *q*-values are not reported in this table. Vitamin B3 refers to niacin-equivalent intake. β, unstandardized regression coefficient; CI, confidence interval; HC3, heteroskedasticity-consistent covariance estimator type 3; MoCA, Montreal Cognitive Assessment; OR, odds ratio; SD, standard deviation.

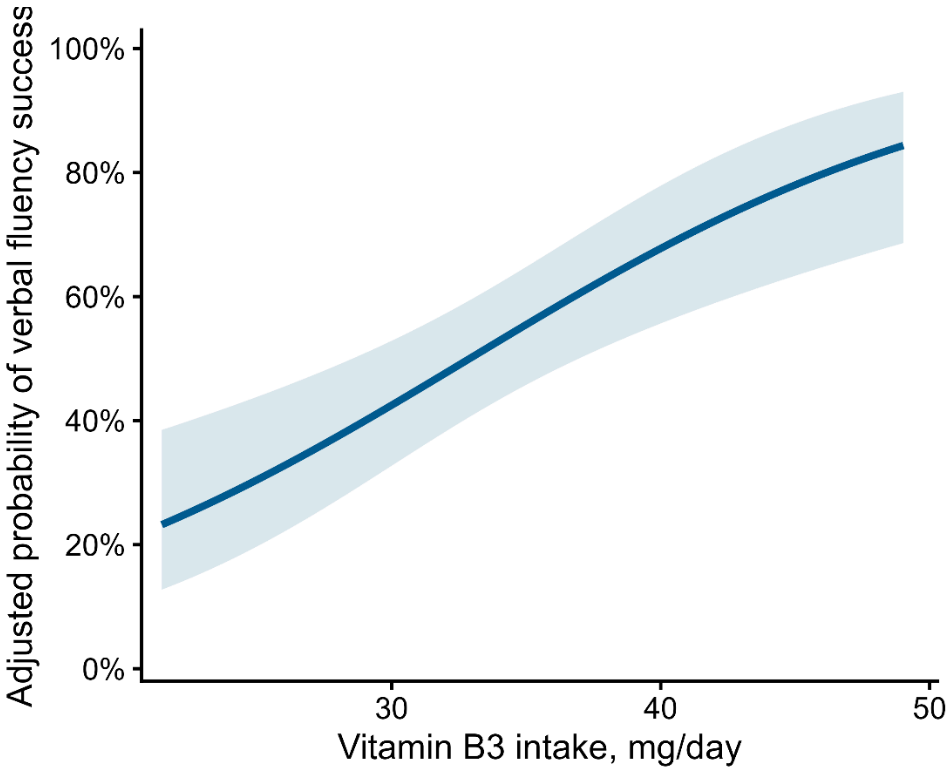

Figure S2. Adjusted association between vitamin B3 intake and verbal fluency. The figure shows the adjusted probability of successful verbal fluency across continuous vitamin B3 intake, estimated from a logistic regression model adjusted for age, sex, educational level, total energy intake, body mass index, and Mediterranean diet adherence. Predictions are displayed across the 5th to 95th percentile range of vitamin B3 intake. The shaded area represents the 95% confidence interval. Vitamin B3 refers to niacin-equivalent intake.

Table S9. Multivariable models of continuous vitamin intake with everyday cognitive complaints and cognitive fatigue.

Outcome	Model	Vitamin	β (95% CI); *p*	FDR *q*-value
MFE-30 total score	Model 1	Vitamin A, per 100 μg RE/day	0.001 (−0.415 to 0.417); *p* = 0.995	0.995
MFE-30 total score	Model 2	Vitamin A, per 100 μg RE/day	0.006 (−0.405 to 0.416); *p* = 0.978	0.995
MFE-30 total score	Model 1	Vitamin B1, mg/day	−1.472 (−6.812 to 3.868); *p* = 0.588	0.995
MFE-30 total score	Model 2	Vitamin B1, mg/day	−0.751 (−6.209 to 4.708); *p* = 0.787	0.995
MFE-30 total score	Model 1	Vitamin B2, mg/day	0.287 (−5.000 to 5.573); *p* = 0.915	0.995
MFE-30 total score	Model 2	Vitamin B2, mg/day	0.863 (−4.579 to 6.304); *p* = 0.755	0.995
MFE-30 total score	Model 1	Vitamin B3, mg/day	0.099 (−0.446 to 0.644); *p* = 0.721	0.995
MFE-30 total score	Model 2	Vitamin B3, mg/day	0.106 (−0.434 to 0.646); *p* = 0.700	0.995
MFE-30 total score	Model 1	Vitamin B6, mg/day	3.445 (−0.237 to 7.128); *p* = 0.067	0.549
MFE-30 total score	Model 2	Vitamin B6, mg/day	3.819 (−0.033 to 7.670); *p* = 0.052	0.549
MFE-30 total score	Model 1	Vitamin B9, μg/day	0.004 (−0.032 to 0.040); *p* = 0.830	0.995
MFE-30 total score	Model 2	Vitamin B9, μg/day	0.004 (−0.031 to 0.039); *p* = 0.834	0.995
MFE-30 total score	Model 1	Vitamin B12, μg/day	0.231 (−0.032 to 0.493); *p* = 0.084	0.549
MFE-30 total score	Model 2	Vitamin B12, μg/day	0.235 (−0.033 to 0.504); *p* = 0.086	0.549

Outcome	Model	Vitamin	β (95% CI); *p*	FDR *q*-value
MFE-30 total score	Model 1	Vitamin C, mg/day	0.004 (−0.040 to 0.047); $p = 0.870$	0.995
MFE-30 total score	Model 2	Vitamin C, mg/day	0.001 (−0.042 to 0.045); $p = 0.962$	0.995
MFE-30 total score	Model 1	Vitamin D, µg/day	−0.035 (−0.884 to 0.815); $p = 0.936$	0.995
MFE-30 total score	Model 2	Vitamin D, µg/day	0.048 (−0.816 to 0.912); $p = 0.913$	0.995
MFIS cognitive subscale	Model 1	Vitamin A, per 100 µg RE/day	−0.058 (−0.249 to 0.132); $p = 0.547$	0.995
MFIS cognitive subscale	Model 2	Vitamin A, per 100 µg RE/day	−0.057 (−0.247 to 0.133); $p = 0.553$	0.995
MFIS cognitive subscale	Model 1	Vitamin B1, mg/day	−0.817 (−3.973 to 2.340); $p = 0.611$	0.995
MFIS cognitive subscale	Model 2	Vitamin B1, mg/day	−0.645 (−3.860 to 2.571); $p = 0.693$	0.995
MFIS cognitive subscale	Model 1	Vitamin B2, mg/day	−0.885 (−3.624 to 1.853); $p = 0.525$	0.995
MFIS cognitive subscale	Model 2	Vitamin B2, mg/day	−0.758 (−3.549 to 2.033); $p = 0.593$	0.995
MFIS cognitive subscale	Model 1	Vitamin B3, mg/day	0.016 (−0.257 to 0.289); $p = 0.907$	0.995
MFIS cognitive subscale	Model 2	Vitamin B3, mg/day	0.018 (−0.256 to 0.292); $p = 0.897$	0.995
MFIS cognitive subscale	Model 1	Vitamin B6, mg/day	1.702 (−0.276 to 3.679); $p = 0.091$	0.549
MFIS cognitive subscale	Model 2	Vitamin B6, mg/day	1.813 (−0.206 to 3.831); $p = 0.078$	0.549
MFIS cognitive subscale	Model 1	Vitamin B9, µg/day	0.013 (−0.006 to 0.032); $p = 0.172$	0.774
MFIS cognitive subscale	Model 2	Vitamin B9, µg/day	0.013 (−0.006 to 0.032); $p = 0.171$	0.774

Outcome	Model	Vitamin	β (95% CI); *p*	FDR *q*-value
MFIS cognitive subscale	Model 1	Vitamin B12, µg/day	0.042 (−0.085 to 0.169); $p = 0.516$	0.995
MFIS cognitive subscale	Model 2	Vitamin B12, µg/day	0.043 (−0.087 to 0.173); $p = 0.514$	0.995
MFIS cognitive subscale	Model 1	Vitamin C, mg/day	0.010 (−0.011 to 0.031); $p = 0.342$	0.995
MFIS cognitive subscale	Model 2	Vitamin C, mg/day	0.010 (−0.011 to 0.030); $p = 0.371$	0.995
MFIS cognitive subscale	Model 1	Vitamin D, µg/day	0.016 (−0.432 to 0.464); $p = 0.944$	0.995
MFIS cognitive subscale	Model 2	Vitamin D, µg/day	0.035 (−0.415 to 0.485); $p = 0.879$	0.995

Values are unstandardized regression coefficients with 95% confidence intervals and unadjusted *p*-values from linear regression models with HC3 robust standard errors. FDR-adjusted *q*-values were calculated using the Benjamini–Hochberg procedure across the 36 self-reported cognition contrasts comprising the MFE-30 total score and MFIS cognitive subscale models. Model-specific sample sizes were 285 for the MFE-30 total score and 283 for the MFIS cognitive subscale in both Model 1 and Model 2. Model 1 was adjusted for age, sex, educational level, and total energy intake. Model 2 was adjusted for age, sex, educational level, total energy intake, body mass index, and Mediterranean diet adherence. Each vitamin was entered as a continuous exposure in a separate model. For vitamin A, coefficients are expressed per 100 µg retinol equivalents/day. Vitamin B3 refers to niacin-equivalent intake. Higher MFE-30 and MFIS cognitive scores indicate greater everyday cognitive complaints and cognitive fatigue, respectively. CI, confidence interval; FDR, false discovery rate; MFE-30, Memory Failures of Everyday Questionnaire; MFIS, Modified Fatigue Impact Scale; RE, retinol equivalents.

Table S10. Sensitivity analyses using MoCA Memory and MoCA Multiple scores.

Outcome	Model	Vitamin	β (95% CI); unadjusted *p*-value	FDR-adjusted *q*-value
MoCA Memory score	Model 1	Vitamin A, per 100 µg RE/day	−0.005 (−0.034 to 0.024); *p* = 0.740	0.987
MoCA Memory score	Model 2	Vitamin A, per 100 µg RE/day	−0.004 (−0.033 to 0.024); *p* = 0.769	0.987
MoCA Memory score	Model 1	Vitamin B1, mg/day	−0.211 (−0.605 to 0.184); *p* = 0.294	0.931
MoCA Memory score	Model 2	Vitamin B1, mg/day	−0.232 (−0.618 to 0.155); *p* = 0.241	0.931
MoCA Memory score	Model 1	Vitamin B2, mg/day	0.003 (−0.362 to 0.368); *p* = 0.987	0.987
MoCA Memory score	Model 2	Vitamin B2, mg/day	0.007 (−0.365 to 0.380); *p* = 0.969	0.987
MoCA Memory score	Model 1	Vitamin B3, mg/day	0.015 (−0.017 to 0.047); *p* = 0.355	0.931
MoCA Memory score	Model 2	Vitamin B3, mg/day	0.013 (−0.018 to 0.045); *p* = 0.410	0.931
MoCA Memory score	Model 1	Vitamin B6, mg/day	−0.120 (−0.340 to 0.100); *p* = 0.288	0.931
MoCA Memory score	Model 2	Vitamin B6, mg/day	−0.117 (−0.341 to 0.107); *p* = 0.306	0.931
MoCA Memory score	Model 1	Vitamin B9, µg/day	0.000 (−0.002 to 0.003); *p* = 0.793	0.987
MoCA Memory score	Model 2	Vitamin B9, µg/day	0.000 (−0.002 to 0.003); *p* = 0.802	0.987
MoCA Memory score	Model 1	Vitamin B12, µg/day	−0.010 (−0.025 to 0.004); *p* = 0.171	0.931
MoCA Memory score	Model 2	Vitamin B12, µg/day	−0.010 (−0.025 to 0.005); *p* = 0.178	0.931
MoCA Memory score	Model 1	Vitamin C, mg/day	−0.001 (−0.004 to 0.001); *p* = 0.298	0.931

Outcome	Model	Vitamin	β (95% CI); unadjusted *p*-value	FDR-adjusted *q*-value
MoCA Memory score	Model 2	Vitamin C, mg/day	−0.001 (−0.004 to 0.001); $p = 0.332$	0.931
MoCA Memory score	Model 1	Vitamin D, μg/day	0.002 (−0.068 to 0.072); $p = 0.955$	0.987
MoCA Memory score	Model 2	Vitamin D, μg/day	−0.004 (−0.068 to 0.061); $p = 0.906$	0.987
MoCA Multiple score	Model 1	Vitamin A, per 100 μg RE/day	−0.004 (−0.024 to 0.016); $p = 0.699$	0.987
MoCA Multiple score	Model 2	Vitamin A, per 100 μg RE/day	−0.004 (−0.025 to 0.017); $p = 0.711$	0.987
MoCA Multiple score	Model 1	Vitamin B1, mg/day	0.179 (−0.302 to 0.660); $p = 0.465$	0.931
MoCA Multiple score	Model 2	Vitamin B1, mg/day	0.146 (−0.333 to 0.626); $p = 0.550$	0.987
MoCA Multiple score	Model 1	Vitamin B2, mg/day	−0.036 (−0.443 to 0.371); $p = 0.862$	0.987
MoCA Multiple score	Model 2	Vitamin B2, mg/day	−0.056 (−0.456 to 0.344); $p = 0.784$	0.987
MoCA Multiple score	Model 1	Vitamin B3, mg/day	0.002 (−0.030 to 0.033); $p = 0.917$	0.987
MoCA Multiple score	Model 2	Vitamin B3, mg/day	0.001 (−0.030 to 0.032); $p = 0.951$	0.987
MoCA Multiple score	Model 1	Vitamin B6, mg/day	−0.169 (−0.368 to 0.029); $p = 0.095$	0.931
MoCA Multiple score	Model 2	Vitamin B6, mg/day	−0.184 (−0.385 to 0.018); $p = 0.075$	0.931
MoCA Multiple score	Model 1	Vitamin B9, μg/day	−0.001 (−0.003 to 0.001); $p = 0.465$	0.931
MoCA Multiple score	Model 2	Vitamin B9, μg/day	−0.001 (−0.003 to 0.001); $p = 0.454$	0.931
MoCA Multiple score	Model 1	Vitamin B12, μg/day	−0.010 (−0.025 to 0.005); $p = 0.175$	0.931

Outcome	Model	Vitamin	β (95% CI); unadjusted *p*-value	FDR-adjusted *q*-value
MoCA Multiple score	Model 2	Vitamin B12, μg/day	−0.010 (−0.026 to 0.005); $p = 0.186$	0.931
MoCA Multiple score	Model 1	Vitamin C, mg/day	−0.001 (−0.004 to 0.002); $p = 0.456$	0.931
MoCA Multiple score	Model 2	Vitamin C, mg/day	−0.001 (−0.004 to 0.002); $p = 0.522$	0.987
MoCA Multiple score	Model 1	Vitamin D, μg/day	0.004 (−0.053 to 0.060); $p = 0.894$	0.987
MoCA Multiple score	Model 2	Vitamin D, μg/day	−0.001 (−0.057 to 0.054); $p = 0.961$	0.987

Values are unstandardized regression coefficients with 95% confidence intervals and unadjusted *p*-values from linear regression models with HC3 robust standard errors. Confidence intervals and p-values were calculated using the t distribution with the residual degrees of freedom of each model. FDR-adjusted *q*-values were calculated using the Benjamini–Hochberg procedure across the 36 MoCA-derived score contrasts. All models included 286 participants. Model 1 was adjusted for age, sex, educational level, and total energy intake; Model 2 additionally included body mass index and Mediterranean diet adherence. Each vitamin was entered separately. Vitamin A coefficients are expressed per 100 μg retinol equivalents/day. Vitamin B3 refers to niacin-equivalent intake. MoCA Memory and MoCA Multiple correspond to raw sums from the item sets defining the 12-item two-factor MoCA solution [44]. The theoretical score ranges were 0–5 for MoCA Memory and 0–9 for MoCA Multiple. CI, confidence interval; FDR, false discovery rate; MoCA, Montreal Cognitive Assessment; RE, retinol equivalents.
